# Probing Cinnamamides and Benzamides as Anthelmintics: Discovery of Potent Drug‐Like Agents Against *Angiostrongylus cantonensis*

**DOI:** 10.1111/cbdd.70353

**Published:** 2026-07-04

**Authors:** Bruna L. Lemes, Mariana A. Siegl‐Breno, Marina T. Varela, Flavia B. Lopes, Mikaelly K. Silva‐Nunes, Thaiane D. Santos, Lucas Fukui‐Silva, Daniel B. Roquini, Vinicius G. Maltarollo, Josué de Moraes, João Paulo S. Fernandes

**Affiliations:** ^1^ Research Center on Neglected Diseases Guarulhos University Guarulhos Brazil; ^2^ Department of Pharmaceutical Sciences Federal University of São Paulo Diadema Brazil; ^3^ Department of Medicine Federal University of São Paulo São Paulo Brazil; ^4^ Research Center on Neglected Diseases, Scientific and Technological Institute Brasil University São Paulo Brazil; ^5^ Department of Pharmaceutical Products, Faculty of Pharmacy Federal University of Minas Gerais Belo Horizonte Brazil

**Keywords:** angiostrongyliasis, anthelmintic agents, drug‐likeness, structure–activity relationships

## Abstract

Emergent helminthiases are increasingly impacting global health in both humans and animals, especially given the limited efficacy of existing drugs against these infections. Neuroangiostrongyliasis, an eosinophilic meningitis caused by *Angiostrongylus cantonensis* , currently lacks effective treatment, highlighting the need for novel anthelmintics. We previously identified cinnamoyl‐benzylpiperazine, a simplified analogue of cinnarizine, as an effective anthelmintic agent against first (L1) and third‐stage (L3) larvae of *A. cantonensis* in vitro. In the present work, structural modifications on the active prototype cinnamoyl‐benzylpiperazine were performed, prioritizing the improvement of solubility and the provision of balanced physicochemical properties compatible with blood–brain barrier permeability, alongside anthelmintic activity. A set of 31 compounds divided into two series (I—cinnamoyl and II—benzoyl) was synthesized and tested against L1 and L3 larvae, yielding EC_50_ values ranging from 4.1 to 27.6 μM. SAR analyses revealed that the activity of set I is strongly associated with balanced electronic density in both the cinnamamide and amine regions (described by ionization potential descriptors), whereas modifications in the charge distribution of the molecules (indicated by topological charge descriptors) appear to determine the anthelmintic activity of set II. None of the compounds displayed significant toxicity to HaCaT mammalian cells (up to 200 μM) or *Caenorhabditis elegans* worms (up to 1000 μM), denoting specific activity against *A. cantonensis* . The balanced polarity of compound **4a‐I** (EC_50_ L1 4.7 μM; L3 10.2 μM) and the localized charge density provided by the methoxy group in compounds **1c‐II** (EC_50_ L1 5.5 μM; L3 10.9 μM) and **5c‐II** (EC_50_ L1 4.9 μM; L3 11.9 μM) seem important for interacting with the putative target in the helminth. Collectively, the substituents in these molecules provided improved drug‐likeness over the previous set of compounds, and represent noteworthy derivatives for further investigation against *A. cantonensis* in vitro.

## Graphical abstract

Cynnamoyl benzylpiperazine was identified as drug‐like derivative of cinnarizine with anthelmintic activity against *A. cantonensis* . In this work, 15 out of 31 derivatives showed improved anthelmintic activity with increased solubility. A SAR study was carried out to determine the molecular features involved in such effect.

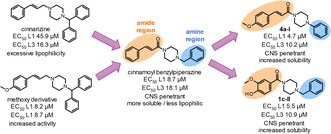

## 1 Introduction

Helminth infections remain among the most prevalent neglected tropical diseases worldwide, disproportionately affecting populations living in conditions of socioeconomic vulnerability with limited access to healthcare. More than one billion people are estimated to be infected with at least one species of parasitic helminth, resulting in substantial morbidity, impaired quality of life, and long‐term socioeconomic consequences (Longo and De Moraes 2025). These infections are closely aligned with several United Nations Sustainable Development Goals (SDGs), highlighting their broad impact on global health, social inequality, and sustainable development (De Moraes and Geary 2020; Kamgno et al. 2025).

Despite this global burden, the therapeutic arsenal available for the treatment of helminthiases remains limited, and innovation in anthelmintic drug development has progressed slowly in recent decades (De Moraes and Geary 2020). This persistent gap represents a major obstacle to achieving international control and elimination targets and has been recognized by the World Health Organization as a critical challenge, leading to the prioritization of new anthelmintic discovery within its roadmap for neglected tropical diseases (Mengarda et al. 2022, 2023; Specht and Keiser 2023). In this scenario, the limited efficacy of existing drugs, together with emerging resistance and suboptimal pharmacokinetic profiles, further underscores the urgent need for new anthelmintic chemotypes (Longo and De Moraes 2025).

Emerging and re‐emerging helminth infections have attracted increasing attention due to their expanding geographic distribution, zoonotic transmission, and the frequent lack of effective treatment options. Among these, *Angiostrongylus cantonensis* , commonly known as the rat lungworm, represents a growing public health concern (Cowie et al. 2022; Gippet et al. 2023; Lv and Zhou 2025). This parasitic nematode is the causative agent of neuroangiostrongyliasis, a neurological disease in humans characterized by eosinophilic meningitis that can result in severe morbidity (Ansdell and Wattanagoon 2018; Cowie et al. 2022). The life cycle of *A. cantonensis* involves rodents as definitive hosts and mollusks as intermediate hosts, with humans acting as accidental hosts following the ingestion of infective larvae (Griffin et al. 2025). Adult worms in rodents release first‐stage larvae (L1), which are shed in feces and subsequently develop into third‐stage larvae (L3) within mollusks. After accidental infection, larvae migrate to the central nervous system, where their presence and subsequent death trigger a pronounced inflammatory response. To date, there is no specific, evidence‐based antiparasitic therapy for neuroangiostrongyliasis, and clinical management relies primarily on corticosteroids to control inflammation, while the use of anthelmintic drugs remains controversial due to inconsistent efficacy (Ansdell et al. 2021). Albendazole, the most commonly used anthelmintic in this context, shows variable clinical benefit and limited penetration into the central nervous system, further complicating therapeutic decision‐making (Ansdell et al. 2021; Lv and Zhou 2025).

**FIGURE 1:**
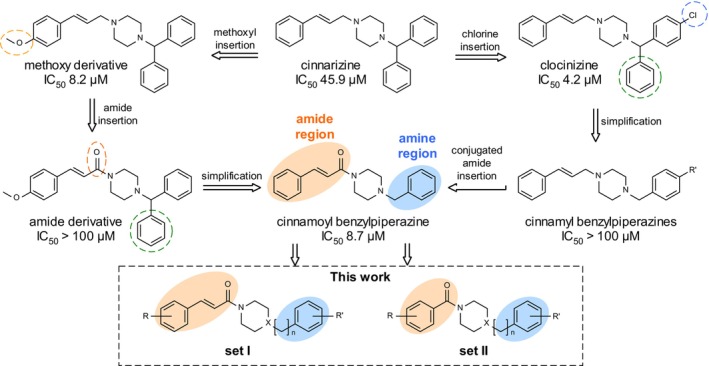
Schematic concept to design the novel derivatives from the sets I and II.

We previously reported the anthelmintic activity of first‐generation H_1_‐antihistamines against *A. cantonensis* larvae (Roquini et al. 2024). Among these drugs, cinnarizine has demonstrated promising properties as a hit compound for further optimization. The analogue clocinizine was tenfold more potent than cinnarizine, and structural exploitation of these drugs led to the identification of the simplified amide analogue of cinnarizine, cinnamoyl benzylpiperazine (Figure 1), which represents a novel chemotype with interesting anthelmintic activity combined with increased solubility and better drug‐likeness than the prototypes (Lemes et al. 2025). However, the activity of these compounds is not correlated with antihistamine activity, and the molecular target for these compounds in the worm remains unknown.

In the present work, we aimed to explore structural modifications on this hit compound through in vitro assays against *A. cantonensis* larvae to further improve its drug‐like profile and elucidate additional structure–activity relationships (SAR) for this chemotype. To this end, we designed two subsets of compounds: the cinnamoyl (set I) and the benzoyl (set II) amides containing benzyl or phenyl groups linked to the cycloamine moiety. These analogues were designed to investigate the role of substituents in both amide and amine regions in modulating anthelmintic activity. The lack of anthelmintic activity for the amine analogue cinnamyl piperazine in previous work clearly denotes the importance of the electronic conjugation between the carbonyl and the aromatic ring in this chemotype; therefore, this motif was maintained in this new set of compounds. In addition, cytotoxicity to mammalian cells, selectivity over other helminths, drug‐like properties, and the predicted ability to cross the blood–brain barrier (a critical requirement for therapeutic intervention in neuroangiostrongyliasis) were monitored when designing these compounds. Finally, classification QSAR studies were conducted to elucidate the impact of these structural modifications on the observed anthelmintic activity.

## 2 Results and Discussion

**SCHEME 1:**
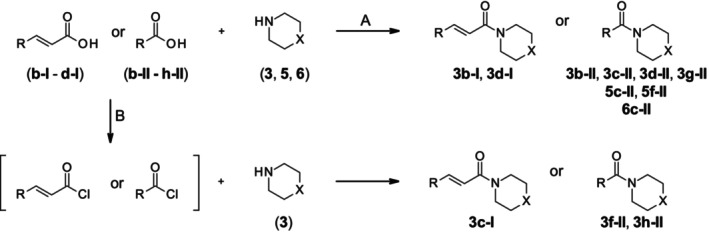
Synthetic scheme for the preparation of the compounds. Reagents and conditions: Reagents and conditions. (**A**) carboxylic acid, EDC.HCl, HOBt.xH_2_O, DCM or DMF, r.t., 1 h then amine, 18–20 h; (**B**) carboxylic acid, SOCl_2_, DCM, 50°C, 1–2 h then amine, TEA, r.t., 2–4 h.

A set of 31 compounds was synthesized following the previously reported method, in which the preparation and characterization of the benzylpiperazine (**1**), benzylpiperidine (**2**), and phenylpiperazine (**4**) compounds are described (Varela et al. 2020; Varela, de Castro Levatti, et al., 2023). The reaction scheme is depicted in Scheme 1. Most compounds were prepared using coupling agents (EDC and HOBt) in DCM (method A); however, when the carboxylic acids were insoluble in DCM (catechol‐containing compounds), DMF was employed as solvent. The limited solubility of the starting materials under these reaction conditions was the main factor affecting yields. Nonetheless, the yields were considered adequate (40%–65%) for pure final compounds after chromatographic purification. The remaining compounds were prepared by converting the carboxylic acids into their corresponding acyl chlorides via in situ reaction with thionyl chloride, followed by the addition of the substituted piperazine derivative in DCM (method B) due to low yields obtained with method A. Considering that most compounds contain hydroxy groups in the carboxylate moiety, conversion to acyl chlorides was avoided due to the possibility of forming phenyl benzoates as byproducts. This is evident in the low yield obtained in the preparation of compound **3f‐II**. In the case of the benzofuran derivative **3 h‐II**, the commercially available acyl chloride was used.

The compounds were tested for their anthelmintic activity against *A. cantonensis* L1 larvae using the methodology previously described by our group (Lemes et al. 2025; Roquini et al. 2024). For those displaying considerable efficacy, the activity against the L3 stage was also assessed. Cytotoxicity toward HaCat cells was evaluated to identify potential non‐selective agents, whereas activity against adult *C. elegans* was used to assess selectivity toward parasitic nematodes, given its established use as a simplified in vivo model. The results are reported in Tables 1 and 2.

**TABLE 1:** In vitro anthelmintic activity and selectivity profile of cinnamoyl piperazine derivatives (set I) against *A. cantonensis* larvae.

ID	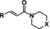		EC_50_ (μM ± SEM)		HaCaT CC_50_ (μM ± SEM)	*C. elegans* LC_50_ (μM ± SEM)
	R	X	L1	L3		
**1a‐I**	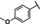	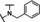	> 100	n.d.	> 200	> 1000
**1b‐I**	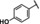	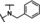	> 100	n.d.	> 200	> 1000
**1c‐I**	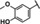	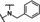	> 100	n.d.	> 200	> 1000
**1d‐I**	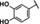	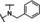	5.1 ± 0.9*	11.3 ± 1.5	> 200	> 1000
**1e‐I**	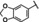	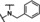	5.0 ± 0.8*	12.8 ± 1.2	> 200	> 1000
**2a‐I**	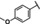	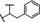	4.7 ± 1.3*	12.4 ± 1.5	> 200	> 1000
**2b‐I**	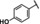	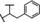	5.1 ± 1.1*	12.3 ± 0.9	> 200	> 1000
**3b‐I**	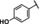	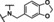	> 100	n.d.	> 200	> 1000
**3c‐I**	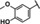	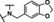	> 100	n.d.	> 200	> 1000
**3d‐I**	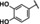	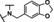	4.1 ± 1.2*	23.3 ± 2.2	> 200	> 1000
**4a‐I**	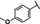	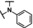	4.7 ± 1.1*	10.2 ± 1.3	> 200	> 1000
**4c‐I**	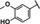	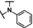	> 100	n.d.	> 200	> 1000
**4d‐I**	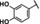	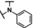	> 100	n.d.	> 200	> 1000
**ALB**			15.4 ± 1.5	16.2 ± 1.4	n.d.	n.d.

*Note:* Data are expressed as mean ± standard error of mean (SEM) from three independent experiments. EC_50_ values were determined by nonlinear regression analysis with 95% confidence intervals.

Abbreviation: ALB, albendazole.

* *p* < 0.01 compared with albendazole for the corresponding larval stage.

**TABLE 2:** In vitro anthelmintic activity and selectivity profile of benzoyl piperazine derivatives (set II) against *A. cantonensis* larvae.

ID	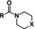		EC_50_ (μM ± SEM)		HaCaT CC_50_ (μM ± SEM)	*C. elegans* EC_50_ (μM ± SEM)
	R	X	L1	L3		
**1a‐II**	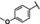	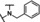	4.6 ± 1.4*	12.5 ± 1.2	> 200	> 1000
**1b‐II**	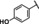	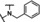	> 100	n.d.	> 200	> 1000
**1c‐II**	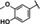	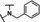	5.5 ± 0.8*	10.9 ± 1.7	> 200	> 1000
**1d‐II**	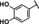	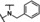	> 100	n.d.	> 200	> 1000
**1 g‐II**	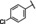	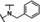	> 100	n.d.	> 200	> 1000
**2a‐II**	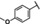	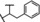	> 100	n.d.	> 200	> 1000
**3b‐II**	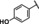	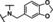	> 100	n.d.	> 200	> 1000
**3c‐II**	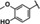	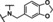	4.6 ± 1.6*	27.6 ± 2.5	> 200	> 1000
**3d‐II**	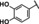	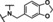	4.1 ± 1.2*	11.3 ± 1.7	> 200	> 1000
**3f‐II**	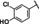	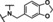	16.7 ± 2.1	25.0 ± 2.7	> 200	> 1000
**3 g‐II**	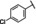	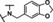	> 100	n.d.	> 200	> 1000
**3 h‐II**	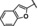	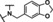	4.2 ± 1.2*	9.9 ± 1.1	> 200	> 1000
**4a‐II**	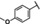	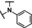	> 100	n.d.	> 200	> 1000
**4c‐II**	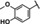	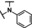	4.9 ± 1.1*	11.8 ± 1.2	> 200	> 1000
**4d‐II**	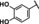	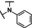	> 100	n.d.	> 200	> 1000
**5c‐II**	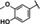	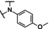	4.9 ± 0.9*	11.9 ± 1.1	> 200	> 1000
**5f‐II**	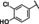	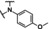	> 100	n.d.	> 200	> 1000
**6c‐II**	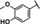	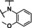	8.2 ± 1.2*	12.5 ± 1.9	> 200	> 1000
**ALB**			15.4 ± 1.5	16.2 ± 1.4	n.d.	n.d.

*Note:* Data are expressed as mean ± standard error of mean (SEM) from three independent experiments. EC_50_ values were determined by nonlinear regression analysis with 95% confidence intervals.

Abbreviation: ALB, albendazole.

* *p* < 0.01 compared with albendazole for the corresponding larval stage.

Within the series, 15 compounds displayed notable activity profiles, with activity comparable or superior to the reference drug, albendazole (ALB). The anthelmintic activity of the compounds (EC_50_ values) ranged from 16.7 to 4.1 μM against L1 larvae, while the activity against L3 larvae varied between 27.6 and 9.9 μM. The most active compounds are 3‐fold more potent than ALB, and showed comparable efficacy to previously described analogues, however with improved drug‐likeness (Table S1). The assessment of drug‐like properties using the SwissADME on‐line platform (Daina et al. 2017) indicated that the solubility of the compounds reported here is superior to that of the previous series (Lemes et al. 2025), which can be attributed to the insertion of polar substituents on both sides of the molecules (Varela et al. 2022; Varela, Romanelli, et al. 2023b). The high drug‐likeness of these compounds is reflected in their quantitative estimate of drug‐likeness (QED) score (Table S1), which considers the contribution of several parameters related to drug‐likeness (Mignani et al. 2018).

The improved balance between the lipophilicity and the polar surface area (determined by the TPSA/WlogP relationship using the BOILED‐Egg model) allows for the prediction of adequate gastrointestinal tract (GIT) absorption for all compounds and, except for compounds **3d‐I**, **3c‐II**, and **3d‐II**, high blood–brain barrier (BBB) permeability (Figure S49). Moreover, compounds containing catechol (**d**) and/or benzodioxole (**e** and **3**) motifs were predicted to be substrates of P‐glycoprotein (P‐gp), indicating that these structural motifs may increase affinity for this counter‐transport protein and compromise their bioavailability and/or BBB permeability (Table S1). Given the CNS involvement in neuroangiostrongyliasis, BBB penetration is considered a critical determinant of therapeutic success (Lemes et al. 2025). In this context, the pharmacokinetic limitations of albendazole, including poor solubility, variable bioavailability, and dependence on metabolic activation, further support prioritizing molecules with higher potential to access the CNS (adequate balance TPSA/WlogP and avoidance of P‐gp substrate pharmacophores).

**FIGURE 2:**
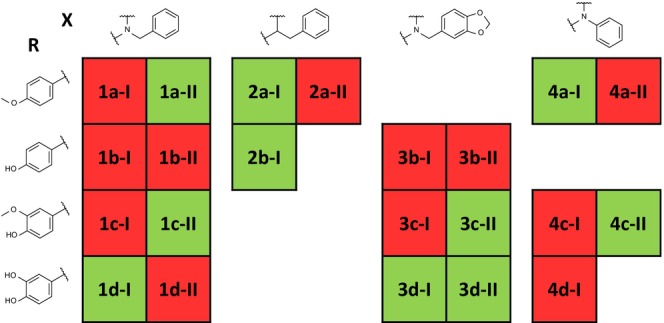
Structure–activity relationships (SAR) matrix for selected compounds. Red, Inactive; Green, Active.

The results clearly indicate that compounds from the sets I and II represent two distinct chemical spaces, as evidenced by pronounced activity cliffs between matched pairs (Figure 2) (Cruz‐Monteagudo et al. 2014). For instance, compounds containing the same motifs in the amine (**1–4**) and amide (**a‐d**) regions for the cinnamoyl (set I) and benzoyl (set II) series displayed opposite activity results. For instance, guaiacol‐containing compounds (**c**) from set I were generally inactive, while the opposite was observed for set II. The SAR matrix (Gupta‐Ostermann and Bajorath 2014) representation from Figure 2 clearly demonstrates this SAR discontinuity in the activity profiles.

**FIGURE 3:**
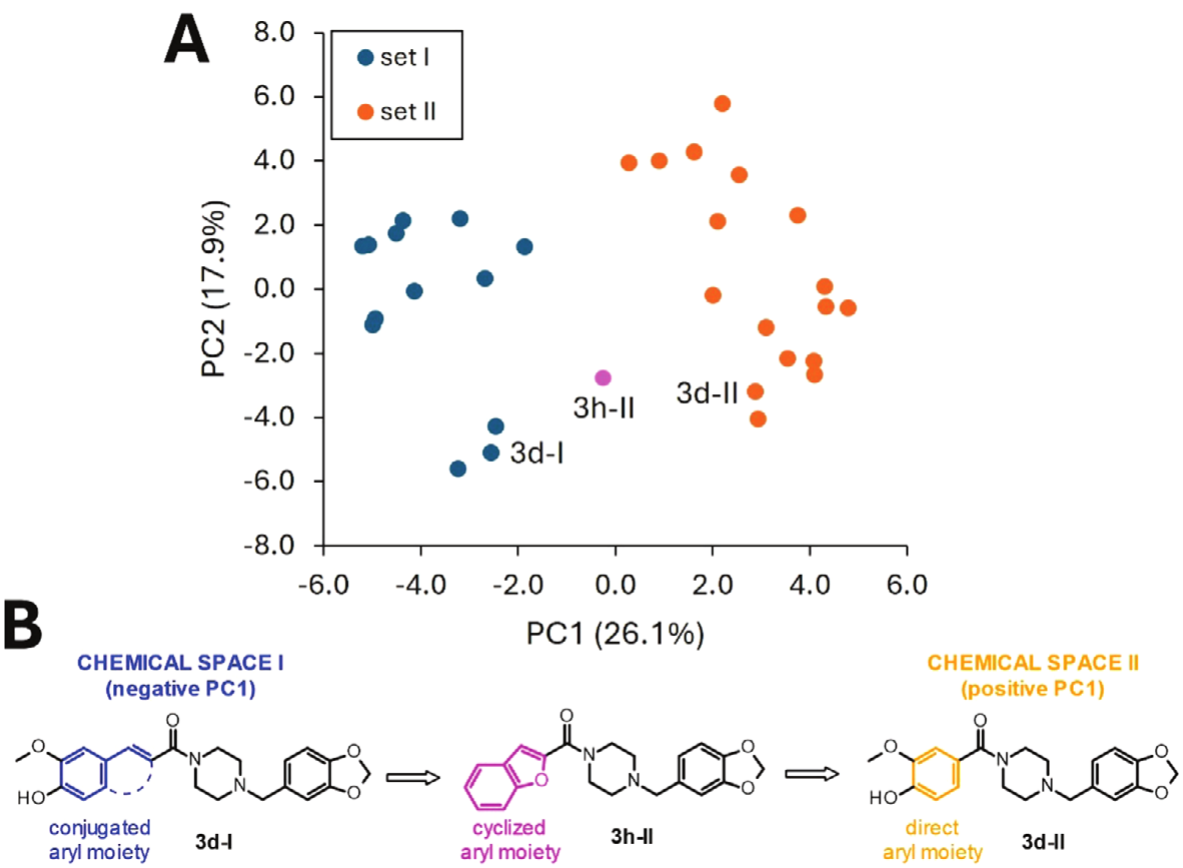
(A) Principal component analysis (PCA) of compounds from sets I (blue) and II (orange) based on AtomPairs fingerprints. The scatterplot shows the distribution of compounds along the first two principal components (PC1 and PC2). Compound **3 h‐II** is highlighted (in magenta) due to its intermediate position between both chemical spaces. (B) Structural scheme depicting the “bridge” made by compound **3 h‐II** between set I (represented by **3d‐I**) and II (represented by **3d‐II**).

A chemometric study using the molecular fingerprints reinforced this hypothesis. As shown in Figure 3A, the scatterplot obtained from a principal component analysis (PCA) using AtomPairs fingerprint values shows a clear separation between the sets I and II along the PC1 axis, delineating their belonging to two distinct chemical spaces. Compounds from set I (cinnamoyls) have negative PC1 values, while the benzoyl derivatives (set II) present positive PC1 values. Interestingly, compound **3 h‐II** has a PC1 value close to zero, positioning it at the boundary between the two sets and suggesting it has mixed characteristics. In fact, the 2‐benzofuranoyl group can be rationalized as a “bridge compound”, since it is a cyclic analogue of the cinnamoyl group (set I) but also contains the benzamide characteristic of set II (Figure 3B), representing the structural hop between the two chemical spaces. The separation of molecules into distinct chemical spaces was also noted using additional fingerprinting methods, reinforcing this conclusion (Figure S50).

**FIGURE 4:**
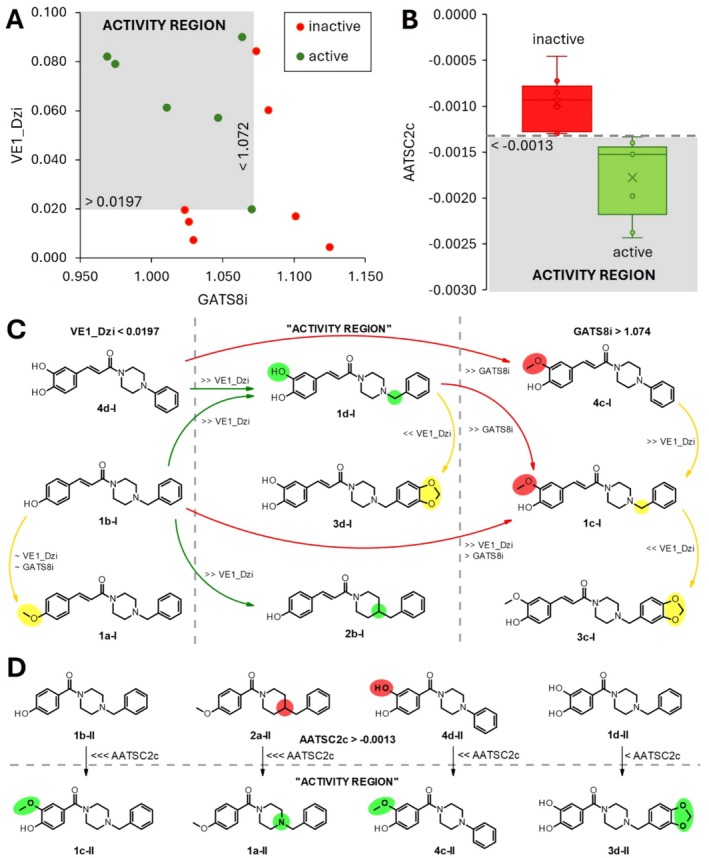
Graphical representation of the selected descriptors classifying the active and inactive compounds from set I and II. (A) Scatterplot of the GATS8i vs. VE1Dzi values for the compounds of set I. (B) Boxplot of the AATSC2c values for the compounds of set II. Schematic SAR representations of matched pairs are displayed in (C) for the set I and in (D) for the set II. Green dots: Active compounds; Red dots: Inactive compounds; Arrows and regions: Green: Modifications that increased the activity; Red: Modifications that decreased the activity; Yellow: Modifications with minor influence on activity.

Since the direct SAR analysis using all the compounds was not straightforward, more sophisticated analyses employing multiple molecular descriptors were conducted. Each subset was submitted to a decision tree method to identify molecular descriptors related to the anthelmintic activity (Figure S51). Relationships were identified between the anthelmintic activity of the compounds from set I and the descriptors VE1_Dzi and GATS8i (Table S2), which are related to the ionization potential (Todeschini and Consonni 2009). Higher VE1_Dzi values showed a positive correlation with anthelmintic activity, whereas inactive compounds presented values lower than 0.0197 (except two inactive compounds **1c‐I** and **3b‐I**). The remaining compounds were correctly classified by the GATS8i descriptor's value, for which the active compounds present GATS8i < 1.072. Although compounds **1c‐I** and **3b‐I** present VE1_Dzi values > 0.0197, they contain methoxy or methylenedioxy substituents that excessively increase GATS8i values to > 1.072, suggesting that electronic density in the amine and amide regions is determinant of the activity (Figure 4A).

The schematic contribution of key substituents in the set I is depicted on Figure 4C. The insertion of a methylene unit between the piperazine and phenyl groups of **4d‐I**, the hydroxylation of **1b‐I** at the 3‐position of the cinnamoyl ring or the modification of its piperazine to piperidine increased the value of the VE1_Dzi descriptor for compounds **1d‐I** and **2b‐I** to > 0.0197 due to a more delocalized electronic distribution. In parallel, the presence of the 3‐methoxy group from the guaiacol moiety (compounds **c**) excessively increased the values for the GATS8i descriptor, denoting that higher electronic dissimilarity at long range (8‐bond) is detrimental to the activity. These data suggest that balanced electronic density in the molecules of set I, described by both VE1_DZi and GATS8i, is important for the interaction with the putative molecular target in the helminth.

For compounds from set II, discrimination between active and inactive compounds was reached using the values for AATSC2c (Figure 4B), a descriptor dependent on variations in the atomic charges at a 2‐bond distance (Todeschini and Consonni 2009). Compounds presenting values lower than −0.0013 for AATSC2c exhibit pronounced charge alternation between neighboring atomic environments, which seems correlated with anthelmintic activity (Table S2). Inactive compounds present higher values (> −0.0013), indicating that changes in the charge distribution compromise the interaction with the putative molecular targets (Figure 4B).

This pattern in set II suggests that anthelmintic activity is favored on molecules exhibiting enhanced local charge heterogeneity, promoting more selective and adaptable short‐range electrostatic interactions within the putative binding site. For example, the active compounds **4c‐II** and **1c‐II** display localized electronic heterogeneity on the methoxy group in the amide region, illustrating the favorable influence of the methylation on the inactive compound **4d‐II**, and the more favorable influence of the methoxy group inserted on the inactive compound **1b‐II** (Figure 4D). This strong local charge heterogeneity is also noted for the active benzylpiperazine **1a‐II** in relation to its inactive counterpart **2a‐II**, explaining why the modification to piperidine was detrimental to the activity. In addition, the presence of the methoxy group (on either the 2‐ or 4‐position) on the amine region of compounds **6c‐II** or **5c‐II** does not significantly influence the activity but considerably increases the solubility of these compounds and balances their lipophilicity in relation to their equipotent analogue **4c‐II**. These modifications are mainly important for tuning the expected pharmacokinetic profile, making compounds that contain guaiacol or methoxyphenyl scaffolds more adequate from this point of view.

**FIGURE 5:**
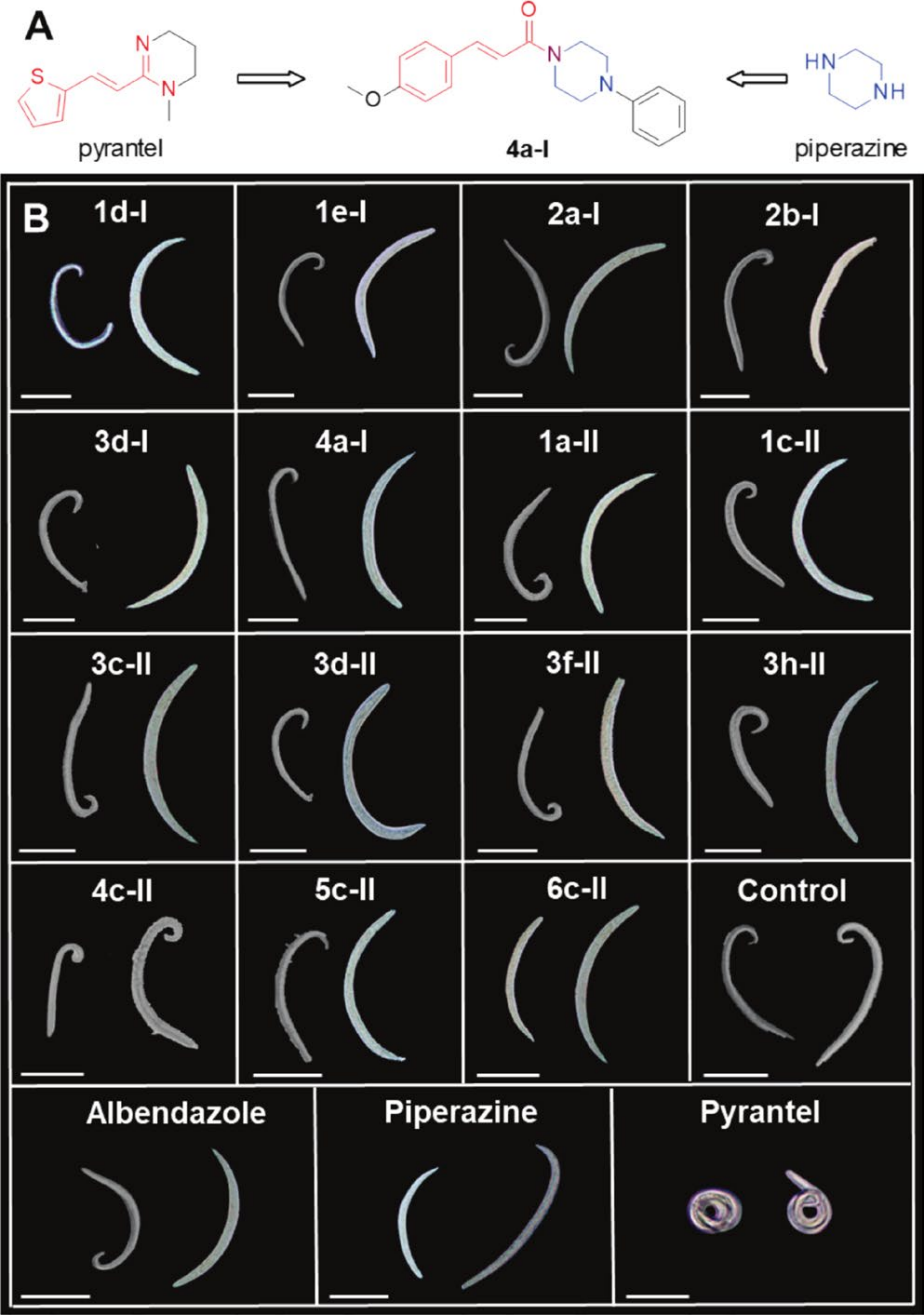
(A) Structural similarities among pyrantel, piperazine, and the compound **4a‐I**. (B) Morphological effects of selected compounds and reference anthelmintics on *A. cantonensis* larvae. Representative images of first‐stage (L1, left) and third‐stage (L3, right) larvae after 24 h of exposure to selected active compounds from sets I and II at concentrations corresponding to twofold their respective EC_50_ values, as determined in Tables 1 and 2. Scale bars = 35 μm.

The compounds reported herein share some structural motifs with two classical anthelmintics: pyrantel and piperazine (Figure 5A). Pyrantel exerts a depolarizing neuromuscular‐blocking effect on the susceptible helminths through sustained activation of cholinergic nicotinic receptors (Rayes et al. 2001), resulting in spastic paralysis. Similar effects were observed in *A. cantonensis* larvae treated with pyrantel (Figure 5B). In contrast, piperazine is a muscular GABA receptor agonist on the worms, inducing a flaccid paralysis and facilitating their expulsion (Del Castillo et al. 1963). Consistent with this pharmacological comparison, exposure of *A. cantonensis* larvae to the active compounds resulted in a flaccid and elongated phenotype, closely resembling the effect induced by piperazine and clearly distinct from the spastic contraction caused by pyrantel (Figure 5B). ALB, used as the reference anthelmintic, produced only modest morphological alterations under the same conditions. In previous work, it was noted that the effects of antihistamines on *A. cantonensis* are not related to their potency at the histamine H_1_ receptor nor to the anticholinergic activity of such compounds (Roquini et al. 2024). However, the exact mechanism of action of such compounds remains elusive and warrants further investigation.

A recent paper (Vairoletti et al. 2022) reported the anthelmintic activity of analogues of fluopyram, a structural analogue of the compounds herein described. Fluopyram and its analogues are supposed to act by binding to the mitochondrial complex II Q‐site (Burns et al. 2015; Chen et al. 2020), leading to anthelmintic effects on both platyhelminths and nemathelminths, thus being considered a pan‐anthelmintic. The high similarity between our compounds and fluopyram analogues sheds light on a novel hypothesis regarding the potential mechanism of action for these compounds that should be investigated in future works.

Overall, the novel chemotype reported here represents drug‐like molecules with notable anthelmintic activity against *A. cantonensis* larvae in vitro. Compounds **4a‐I**, **1c‐II**, and **5c‐II** are highlighted due to their balanced solubility/lipophilicity providing adequate drug‐likeness allied to elevated potency. Although these findings provide strong in vitro and *in silico* support for these compounds as promising prototypes, in vivo studies will be required to confirm efficacy, pharmacokinetics, CNS exposure, and safety. Collectively, these compounds define two novel chemotypes that may serve as prototypes for further designing effective anthelmintic agents against this emergent parasite.

## 3 Material and Methods

### 3.1 Reagents and Equipment

The starting materials and chemicals were purchased from LabSynth (Diadema, SP, Brazil) and Sigma‐Aldrich (Saint Louis, MO, USA) at adequate purity level for the procedures and were used without any further purification. Compounds containing benzylpiperazine (**1**), benzylpiperidine (**2**), and phenylpiperazine (**4**) were prepared and characterized as previously reported by our group (Varela, de Castro Levatti, et al. 2023a).

^1^H and ^13^C NMR spectra were recorded on a Bruker Ultrashield 300 spectrometer, operating at 300 and 75 MHz, respectively, using CDCl_3_ or DMSO‐*d*_6_ as solvents and tetramethylsilane (TMS) as internal standard (Figures S1–S24). Chemical shifts (δ) were determined from TMS and are reported in parts per million (ppm). Coupling constants (*J*) are reported in units of Hertz (Hz), where applicable. All NMR data were obtained using the compounds as free bases. HRMS analysis was performed using a micrOTOF‐Q ESI‐Qq‐TOF mass spectrometer (Bruker Daltonics, Bremen, Germany) (Figures S25–S36). The purity of the compounds was chromatographically determined on a Shimadzu LC‐20AT HPLC‐UV equipment using a C18 column (ACE‐5) and acetonitrile/H_2_O (70:30) as eluent, with detection at 262 nm wavelength and flow rate of 1.0 mL/min (Figures S37–S48). All the final compounds showed purity level > 95% and were considered adequate for the biological assays.

### 3.2 General Method for Synthesis of Compounds

*Method A*. To a solution of the appropriate carboxylic acid (1.1 mmol) dissolved in 20 mL of dichloromethane (DCM) or 10 mL of *N*,*N*‐dimethylformamide (DMF), 1 equivalent of *N*‐ethyl‐*N*′‐(3‐dimethylaminopropyl)carbodiimide hydrochloride (EDC.HCl) and 1‐hydroxybenzotriazole hydrate (HOBt.xH_2_O) were added and the mixture was stirred for 1 h at room temperature. Afterwards, the appropriate amine derivative (1 mmol) was added. The reaction mixture was stirred at room temperature overnight and then concentrated under reduced pressure. The residue was taken up into 15 mL of DCM and washed twice with NaHCO_3_ solution and water. The organic layer was dried with anhydrous Na_2_SO_4_ and evaporated. The compounds were purified through silica gel column using DCM:MeOH as eluent.

(2E)‐1‐{4‐[(2H‐1,3‐benzodioxol‐5‐yl)methyl]piperazin‐1‐yl}‐3‐(4‐hydroxyphenyl)prop‐2‐en‐1‐one (**3b‐I**): Reaction between 4‐coumaric acid (**b‐I**) and 1‐piperonylpiperazine (**3**) yielded 0.172 g (47%) of a white solid; R_f_ 0.60 (DCM:MeOH 9:1); ^1^H NMR (300 MHz, DMSO‐d_6_) δ 9.85 (s, 1H), 7.52 (d, *J* = 8.4 Hz, 2H), 7.40 (d, *J* = 15.3 Hz, 1H), 6.99 (d, *J* = 15.3 Hz, 1H), 6.91–6.81 (m, 2H), 6.81–6.70 (m, 3H), 5.98 (s, 2H), 3.65 (br.s, 2H), 3.55 (br.s, 2H), 3.41 (s, 2H), 2.34 (br.s, 4H). ^13^C NMR (75 MHz, DMSO‐d_6_) δ165.29, 159.38, 147.68, 146.67, 142.30, 132.01, 130.22, 126.66, 122.57, 116.04, 114.73, 109.62, 108.29, 101.23, 61.96, 53.44, 52.66, 45.49, 42.10. HRMS (ESI+) *m/*z [M + H]^+^ calcd.: 367.1652; [M + H]^+^ found: 367.1660. HPLC Rt 2.41 min.

(E)‐1‐[4‐(1,3‐benzodioxol‐5‐ylmethyl)piperazin‐1‐yl]‐3‐(3,4‐dihydroxyphenyl)prop‐2‐en‐1‐one (**3d‐I**): Reaction between caffeic acid (**d‐I**) and 1‐piperonylpiperazine (**3**) yielded 0.152 g (40%) of a white solid; R_f_ 0.64 (DCM:MeOH 9:1); ^1^H NMR (300 MHz, DMSO‐d_6_) δ 9.43 (s, 1H), 8.97 (s, 1H), 7.31 (d, *J* = 15.1 Hz, 1H), 7.07 (d, *J* = 1.7 Hz, 1H), 6.97 (dd, *J* = 8.3, 1.7 Hz, 1H), 6.91–6.62 (m, 5H), 5.99 (s, 2H), 3.65 (br.s, 2H), 3.55 (br.s, 2H), 3.41 (s, 2H), 2.34 (br.s, 4H). ^13^C RMN (75 MHz, DMSO) δ 165.22, 147.81, 147.69, 146.68, 145.87, 142.65, 132.11, 127.17, 122.53, 121.08, 116.03, 115.30, 114.69, 109.60, 108.32, 101.25, 61.96, 52.67. HRMS (ESI+) *m/*z [M + H]^+^ calcd.: 383.1602; [M + H]^+^ found: 383.1608. HPLC Rt 2.46 min.

4‐{4‐[(2H‐1,3‐benzodioxol‐5‐yl)methyl]piperazine‐1‐carbonyl}phenol (**3b‐II**): Reaction between 4‐hydroxybenzoic acid (**b‐II**) and 1‐piperonylpiperazine (**3**) yielded 0.205 g (60%) of a white solid; R_f_ 0.35 (DCM:MeOH 15:1); ^1^H NMR (300 MHz, CDCl_3_) δ 7.22 (d, *J* = 8.4 Hz, 2H), 6.84 (s, 1H), 6.78–6.66 (m, 4H), 5.94 (s, 2H), 3.71 (br.s, 2H), 3.54 (m, 2H), 3.44 (s, 2H), 2.44 (br.s, 4H). ^13^C NMR (75 MHz, CDCl_3_) δ 170.84, 157.92, 147.75, 146.81, 131.37, 129.17, 126.85, 122.26, 115.39, 109.41, 107.94, 100.95, 62.60. HRMS (ESI+) *m/*z [M + H]^+^ calcd.: 341.1496; [M + H]^+^ found: 341.1499. HPLC R_T_ 2.16 min.

4‐{4‐[(2H‐1,3‐benzodioxol‐5‐yl)methyl]piperazine‐1‐carbonyl}‐2‐methoxyphenol (**3c‐II**): Reaction between vanillic acid (**c‐II**) and 1‐piperonylpiperazine (**3**) yielded 0.186 g (50%) of a yellowish solid. R_f_ 0.35 (DCM/MeOH 20:1); ^1^H NMR (300 MHz, CDCl_3_) δ 7.00 (s, 1H), 6.96–6.87 (m, 2H), 6.85 (s, 1H), 6.80–6.68 (m, 2H), 5.94 (s, 2H), 3.89 (s, 3H), 3.65 (br.s, 4H), 3.44 (s, 2H), 2.44 (br.s, 4H). ^13^C NMR (75 MHz, CDCl_3_) δ 170.25, 147.74, 147.19, 146.80, 146.62, 131.38, 127.45, 122.26, 120.76, 113.90, 110.66, 109.41, 107.93, 100.95, 62.62, 56.01, 52.92. HRMS (ESI+) *m/*z [M + H]^+^ calcd.: 371.1602; [M + H]^+^ found: 371.1606. HPLC R_T_ 2.15 min.

[4‐(1,3‐benzodioxol‐5‐ylmethyl)piperazin‐1‐yl]‐(3,4‐dihydroxyphenyl)methanone (**3d‐II**): Reaction between protocatechuic acid (**d‐II**) and 1‐piperonylpiperazine (**3**) yielded 0.196 g (55%) of a white solid; R_f_ 0.25 (DCM:MeOH 20:1); ^1^H NMR (300 MHz, CDCl_3_) δ 6.82 (d, *J* = 6.0 Hz, 2H), 6.75–6.66 (m, 4H), 6.25 (br.s., 2H), 5.93 (s, 2H), 3.71 (br.s, 2H), 3.47 (br.s, 2H), 3.42 (s, 2H), 2.42 (br.s, 4H). ^13^C NMR (75 MHz, CDCl_3_) δ 171,40, 147,75, 146,96, 146,90, 144,49, 130,84, 126,03, 122,48, 119,56, 114,98, 114,84, 109,54, 107,98, 100,98, 62,46. HRMS (ESI+) *m/*z [M + H]^+^ calcd.: 357.1445; [M + H]^+^ found: 357.1451. HPLC Rt 1.60 min.

1‐[(2H‐1,3‐benzodioxol‐5‐yl)methyl]‐4‐(4‐chlorobenzoyl)piperazine (**3 g‐II**): Reaction between 4‐chlorobenzoic acid (**g‐II**) and 1‐piperonylpiperazine (**3**) yielded 0.196 g (55%) of a white solid; R_f_ 0.70 (DCM:MeOH 15:1); ^1^H NMR (300 MHz, CDCl_3_) δ 7.43–7.30 (m, 4H), 6.84 (s, 1H), 6.80–6.66 (m, 2H), 5.93 (s, 2H), 3.76 (br.s, 2H), 3.43 (s, 2H), 3.43–3.34 (br.s, 2H), 2.42 (br.s, 4H). ^13^C NMR (75 MHz, CDCl_3_) δ 169.17, 147.75, 146.79, 135.70, 134.19, 131.40, 128.73, 128.63, 122.19, 109.34, 107.93, 100.96, 62.57, 53.07, 52.60, 47.75, 42.25. HRMS (ESI+) *m/*z [M + H]^+^ calcd.: 359.1157; [M + H]^+^ found: 359.1161. HPLC Rt 4.51 min.

2‐methoxy‐4‐[4‐(4‐methoxyphenyl)piperazine‐1‐carbonyl]phenol (**5c‐II**): Reaction between vanillic acid (**c‐II**) and 1‐(4‐methoxyphenyl)piperazine (**5**) yielded 0.150 g (43%) of a yellowish solid; R_f_ 0.44 (DCM:MeOH 15:1); ^1^H NMR (300 MHz, CDCl_3_) δ 7.04 (d, *J* = 1.6 Hz, 1H), 6.99–6.74 (m, 6H), 5.87 (br.s, 1H), 3.92 (s, 3H), 3.78 (s, 3H), 3.77 (br.s, 4H), 3.08 (br.s, 4H). ^13^C NMR (75 MHz, CDCl_3_) δ 170.36, 154.43, 147.27, 146.63, 145.28, 127.31, 120.83, 118.93, 114.54, 113.90, 110.64, 56.05, 55.56, 51.12. HRMS (ESI+) *m/*z [M + H]^+^ calcd.: 343.1652; [M + H]^+^ found: 343.1658. HPLC R_T_ 1.99 min.

2‐chloro‐4‐[4‐(4‐methoxyphenyl)piperazine‐1‐carbonyl]phenol (**5f‐II**): Reaction between 3‐chloro‐4‐hydroxybenzoic acid (**f‐II**) and 1‐(4‐methoxyphenyl)piperazine (**5**) yielded 0.221 g (64%) of a yellowish solid; R_f_ 0.4 (DCM/MeOH 20:1); ^1^H NMR (300 MHz, CDCl_3_) δ 7.46 (d, *J* = 2.0 Hz, 1H), 7.25 (dd, *J* = 8,4, 2,0 Hz, 1H), 7.00 (d, *J* = 8,4 Hz, 1H), 6.96–6.74 (m, 4H), 3.78 (br.s, 4H), 3.77 (s, 3H), 3.08 (br.s, 4H). ^13^C NMR (75 MHz, CDCl_3_) δ 169.22, 154.54, 153.36, 145.11, 131.10, 128.84, 128.14, 127.68, 120.39, 119.11, 119.05, 116.41, 114.58, 55.57, 51.25. HRMS (ESI+) *m/*z [M + H]^+^ calcd.: 347.1157; [M + H]^+^ found: 347.1163. HPLC R_T_ 2.34 min.

*2*‐methoxy‐4‐[4‐(2‐methoxyphenyl)piperazine‐1‐carbonyl]phenol (**6c‐II**): Reaction between vanillic acid (**c‐II**) and 1‐(2‐methoxyphenyl)piperazine (**6**) yielded 0.224 g (65%) of a yellowish solid; R_f_ 0.40 (DCM/MeOH 50:1); ^1^H NMR (300 MHz, CDCl_3_) δ 7.13–6.99 (m, 2H), 7.00–6.80 (m, 5H), 3.93 (s, 3H), 3.88 (s, 3H), 3.87 (br.s, 4H), 3.07 (br.s, 4H). ^13^C NMR (75 MHz, CDCl_3_) δ 170.32, 152.28, 147.21, 146.63, 140.70, 127.46, 123.57, 121.07, 120.83, 118.46, 113.89, 111.36, 110.67, 56.04, 55.43, 50.96. HRMS (ESI+) *m/*z [M + H]^+^ calcd.: 343.1652; [M + H]^+^ found: 343.1658. HPLC R_T_ 2.08 min.

*Method B*. To a solution of the carboxylic acid (1 mmol) in 15 mL of DCM, 0.15 mL of thionyl chloride was added and the mixture was stirred at 50°C for 2 h. The resulting solution was evaporated to remove the excess of thionyl chloride and redissolved in 15 mL of DCM, whereupon 1 equivalent of the amine derivative and triethylamine (TEA) were added. The mixture was stirred at room temperature for 2–4 h and afterwards washed thrice with NaHCO_3_ solution and water. The organic layer was dried with anhydrous Na_2_SO_4_ and evaporated. The compounds were purified through silica gel column using DCM:MeOH as eluent.

(2E)‐1‐{4‐[(2H‐1,3‐benzodioxol‐5‐yl)methyl]piperazin‐1‐yl}‐3‐(4‐hydroxy‐3‐metoxyphenyl)prop‐2‐en‐1‐one (**3c‐I**): Reaction between ferulic acid (**c‐I**) and 1‐piperonylpiperazine (**3**) yielded 0.217 g (55%) of a white solid; R_f_ 0.75 (DCM:MeOH 15:1); ^1^H NMR (300 MHz, CDCl_3_) δ 7.60 (d, *J* = 15.3 Hz, 1H), 7.08 (dd, *J* = 8.2, 1.6 Hz, 1H), 6.98 (d, *J* = 1.6 Hz, 1H), 6.95–6.81 (m, 2H), 6.79–6.64 (m, 3H), 5.95 (s, 2H), 3.91 (s, 3H), 3.72 (br.s, 2H), 3.67 (br.s, 2H), 3.44 (s, 2H), 2.51–2.39 (m, 4H). ^13^C RMN (75 MHz, CDCl_3_) δ 165.67, 147.74, 147.39, 146.78, 146.76, 142.93, 131.48, 127.83, 122.25, 121.87, 114.80, 114.49, 109.91, 109.42, 107.93, 100.95, 62.60, 55.98, 53.20, 52.66, 45.87, 42.20. HRMS (ESI+) *m/*z [M + H]^+^ calcd.: 397.1758; [M + H]^+^ found: 397.1763. HPLC Rt 2.53 min.

[4‐(1,3‐benzodioxol‐5‐ylmethyl)piperazin‐1‐yl]‐(3‐chloro‐4‐hydroxy‐phenyl)methanone (**3f‐II**): Reaction between 3‐chloro‐4‐hydroxybenzoic (**f‐II**) acid and 1‐piperonylpiperazine (**3**) yielded 0.112 g (30%) of a white solid; R_f_ 0.51 (DCM/MeOH 15:1); ^1^H NMR (300 MHz, CDCl_3_) δ 7.43 (d, *J* = 2.0 Hz, 1H), 7.22 (dd, *J* = 8.3, 2.0 Hz, 1H), 7.00 (d, *J* = 8.3 Hz, 1H), 6.85 (s, 1H), 6.78–6.69 (m, 2H), 5.95 (s, 2H), 3.55 (br.s, 4H), 3.44 (s, 2H), 2.44 (br.s, 4H). ^13^C NMR (75 MHz, DMSO) δ 209.50, 189.36, 168.21, 154.85, 147.70, 146.69, 132.10, 129.60, 127.89, 127.85, 122.49, 119.95, 116.63, 109.56, 108.31, 101.25, 61.96, 52.79. HRMS (ESI+) *m/*z [M + H]^+^ calcd.: 375.1106; [M + H]^+^ found: 375.1112. HPLC Rt 2.53 min.

1‐[(2H‐1,3‐benzodioxol‐5‐yl)methyl]‐4‐(1‐benzofuran‐2‐carbonyl)piperazine (**3 h‐II**): Reaction between 2‐benzofurancarbonyl chloride (**h‐II**) and 1‐piperonylpiperazine (**3**) yielded 0.300 g (82%) of a white solid; R_f_ 0.90 (DCM: MeOH 15:1); ^1^H NMR (300 MHz, CDCl_3_) δ 7.64 (d, *J* = 7.7 Hz, 1H), 7.51 (dd, *J* = 8.3, 0.6 Hz, 1H), 7.42–7.32 (m, 1H), 7.33–7.22 (m, 2H), 6.87 (s, 1H), 6.75 (s, 2H), 5.95 (s, 2H), 3.85 (br.s, 4H), 3.46 (s, 2H), 2.55–2.49 (m, 4H). ^13^C RMN (75 MHz, CDCl_3_) δ 159.71, 154.59, 149.11, 147.77, 146.81, 141.78, 131.47, 126.99, 126.41, 123.57, 122.25, 122.21, 111.91, 111.86, 109.40, 107.95, 100.96, 62.58, 52.96, 46.72, 43.14. HRMS (ESI+) *m/*z [M + H]^+^ calcd.: 365.1496; [M + H]^+^ found: 365.1501. HPLC Rt 4.70 min.

### 3.3 Parasites and Animal Maintenance

The life cycle of *Angiostrongylus cantonensis* (NPDN‐AC strain) was maintained under laboratory conditions at the Research Center on Neglected Diseases, Guarulhos University. Wistar rats ( *Rattus norvegicus* ) were used as definitive hosts, while freshwater snails ( *Biomphalaria glabrata* ) or giant African land snails ( *Achatina fulica* ) served as intermediate hosts (Teixeira et al. 2025). Animals were kept under controlled environmental conditions (22°C ± 1°C; 50%–60% relative humidity) with free access to food and water.

### 3.4 In Vitro Anthelmintic Activity Assays

First‐stage larvae (L1) of *A. cantonensis* were recovered from fecal material of infected Wistar rats using the Rugai sedimentation technique (Roquini et al. 2022). Larvae were subsequently washed three times with RPMI 1640 medium containing penicillin (100 U/mL) and streptomycin (100 μg/mL). Approximately 50 larvae were transferred to each well of 96‐well plates containing a final volume of 200 μL of supplemented RPMI medium (Teixeira et al. 2025).

For assays using infective third‐stage larvae (L3), larvae were obtained from experimentally infected *A. fulica* snails following enzymatic digestion with pepsin‐HCl (1% pepsin in 0.7% HCl) at 37°C for 2 h, followed by Rugai sedimentation (Fukui‐Silva et al. 2025). After recovery, approximately 50 larvae were dispensed into each well of 96‐well plates containing RPMI medium.

Test compounds were prepared as stock solutions in dimethyl sulfoxide (DMSO) and serially diluted in culture medium to obtain final concentrations starting at 100 μM, with the final DMSO concentration not exceeding 0.5% (v/v). Plates were incubated at 21°C for 24 h. Larval motility was evaluated at 0 and 24 h using an inverted light microscope (Motic, Canada, model AE2000) and classified into four categories: immobile, intermittent movement, slow movement, or high motility (Cirino et al. 2025). Images were captured using the Motic Images Plus 3.0 software (Motic). Compounds were considered active when at least 60% of larvae exhibited complete immobility (Lemes et al. 2025; Roquini et al. 2024). Half‐maximal effective concentrations (EC_50_) were determined by nonlinear regression analysis. All experiments were performed in triplicate and independently repeated three times. Albendazole (ALB) was used as the positive control, while piperazine and pyrantel were included as reference anthelmintics for comparative purposes.

### 3.5 Toxicity to *Caenorhabditis elegans*

The free‐living nematode *C. elegans* (Bristol N2 strain) was maintained on nematode growth medium (NGM) agar plates seeded with *Escherichia coli* OP50 as a food source, according to standard laboratory procedures (Mengarda et al. 2025). To assess nonspecific nematode toxicity, synchronized fourth‐stage (L4) *C. elegans* larvae were used as a counter‐screen. Approximately 60 larvae were placed into each well of 96‐well plates containing M9 buffer. Compounds were evaluated at concentrations up to 1000 μM. Albendazole was used as a positive control, while DMSO (0.5%, v/v) served as the vehicle control (Souza et al. 2024).

After incubation for 24 h at 21°C, larval viability was determined based on motility. Larvae that failed to respond to gentle mechanical stimulation were classified as nonviable (Cirino et al. 2025). Toxicity was defined as ≥ 60% immobility, and EC_50_ values were calculated by nonlinear regression (Fukui‐Silva et al. 2025). Assays were carried out in triplicate and repeated in three independent experiments.

### 3.6 Cytotoxicity Assays

Cytotoxicity was evaluated using immortalized human keratinocytes (HaCaT). Cells were cultured in Dulbecco's Modified Eagle Medium (DMEM) supplemented with 10% fetal bovine serum, penicillin (100 U/mL), and streptomycin (100 μg/mL) at 37°C in a humidified atmosphere containing 5% CO_2_. Cells were seeded into 96‐well plates at a density of 2 × 10^3^ cells per well and exposed to test compounds at concentrations starting from 500 μM. DMSO (0.5%, v/v) was used as the negative control (Sessa et al. 2020).

After 72 h of incubation, cell viability was assessed using the MTT assay (Morais et al. 2021). Briefly, MTT solution was added to each well and incubated for an additional 3 h, after which absorbance was measured at 595 nm using an Epoch microplate spectrophotometer (BioTek Instruments, Winooski, VT, USA). Experiments were performed in triplicate and repeated three times. Half‐maximal cytotoxic concentrations (CC_50_) were calculated by nonlinear regression analysis (Cirino et al. 2025).

### 3.7 In Silico Drug‐Likeness Assessment

The compounds were evaluated for their drug‐like properties using the SwissADME on‐line platform (Daina et al. 2017), where their structures were built and used to calculate a number of physicochemical descriptors, including the lipophilicity (logP) and solubility (logS) values, total polar surface area (TPSA), fraction of sp3 carbons (Csp3), rotatable bonds (RB), hydrogen‐bond donors (HBD) and acceptors (HBA) counts, molar refractivity (MR), and skin permeability (logKp), among others. The results are presented in table S1. The BOILED‐Egg model (Daina and Zoete 2016) implemented in the platform uses the WLogP and TPSA values to estimate the permeability of the compounds through the gastrointestinal tract (GIT) and blood–brain barrier (BBB) (Figure S49). A predictive model to estimate the potential as a substrate of P‐glycoprotein is also implemented in the platform.

### 3.8 Chemometric Analysis

For the SAR analysis, the compounds were classified into active (EC_50_ < 50 μM) and inactive (EC_50_ > 50 μM) groups. The structures of the compounds were built up in the Marvin software (Chemaxon Inc., version 23.17.0) and converted into their canonical SMILES code. The set of compounds was used to calculate molecular descriptors (1D and 2D) using the PaDEL descriptor tool (Yap 2011) and fingerprints using the cheminformatics nodes from the RDKit (Landrum, n.d.) implemented in KNIME workflow environment (Fillbrunn et al. 2017). A preliminary analysis of the resulting data table was carried out to remove descriptors with missing or non‐informative (low variance) values and the resulting descriptors were used in the chemometric analysis.

The AtomPairs, RDKit and Layered fingerprints were generated using their default parameter settings and used in independent principal component analyses (PCA). The resulting PCs were used to generate the plots presented in Figures 3 and S39. The accumulated variances for the main PCs (PC1 and PC2) are depicted in each axis. The use of multiple fingerprint representations enabled the capture of complementary structural features according to previously reported strategies (De Almeida Marques et al. 2024; Pereira et al. 2026), encompassing both local substructural patterns and more global topological relationships, thereby providing a robust and comprehensive assessment of the chemical diversity within the dataset.

Each separated subset of compounds (set I and II) was submitted to Decision Tree method (Song and Lu 2015) to classify the compounds into active and inactive groups using the obtained molecular descriptors calculated in the PaDEL using the default parameters implemented in KNIME (Figure S51 and Table S2). For series I, the selected descriptors were VE1_Dzi (First Eigenvalue of the Distance/Adjacency Matrix weighted by atomic Ionization Potential) and GATS8i (Geary Autocorrelation of Topological Structure at lag 8 weighted by atomic ionization potential) for activity class discrimination (Todeschini and Consonni 2009). These descriptors have no important intercorrelation (R = −0.386). For subset II, only the topological descriptor AATSC2c (Average Centered Topological Autocorrelation at lag 2 weighted by atomic charges) was selected for discrimination between the activity classes (Todeschini and Consonni 2009). A graphical representation of the data obtained by decision trees is displayed in the Figure 4.

## Author Contributions

**Bruna L. Lemes:** data curation, writing – original draft, investigation, methodology, validation, formal analysis. **Lucas Fukui‐Silva:** investigation, data curation, validation, methodology, formal analysis, writing – original draft. **Mariana A. Siegl‐Breno:** investigation, writing – original draft, data curation, methodology, formal analysis, validation. **Daniel B. Roquini:** investigation, data curation, validation, methodology, writing – original draft, formal analysis. **Mikaelly K. Silva‐Nunes:** investigation, data curation, writing – original draft, methodology, validation, formal analysis. **Marina T. Varela:** investigation, data curation, methodology, validation, formal analysis, writing – original draft. **Flavia B. Lopes:** investigation, data curation, methodology, validation, writing – original draft, formal analysis. **Josué de Moraes:** supervision, conceptualization, funding acquisition, writing – original draft, writing – review and editing, project administration, validation, formal analysis, resources, data curation. **Vinicius G. Maltarollo:** methodology, writing – original draft, writing – review and editing, data curation, formal analysis, investigation, resources. **João Paulo S. Fernandes:** conceptualization, funding acquisition, writing – original draft, writing – review and editing, validation, supervision, project administration, data curation, resources, formal analysis. **Thaiane D. Santos:** investigation, data curation, formal analysis, validation, methodology, writing – original draft.

## Funding

This work was supported by Fundação de Amparo à Pesquisa do Estado de São Paulo, 2023/17755‐6, 2023/03485‐7, 2025/18855‐0. Coordenação de Aperfeiçoamento de Pessoal de Nível Superior, 001. Conselho Nacional de Desenvolvimento Científico e Tecnológico, 401169/2025‐1, 312211/2021‐0, 302348/2025‐5.

## Ethics Statement

All experimental procedures involving animals were approved by the Committee for the Ethical Use of Animals in Experimentation at Guarulhos University (Guarulhos, SP, Brazil) under protocol number 064/24. All experiments were conducted in accordance with Brazilian regulations for animal experimentation and complied with applicable ethical guidelines.

## Conflicts of Interest

The authors declare no conflicts of interest.

## Supporting information


**Figure S1:** cbdd70353‐sup‐0001‐TableS1‐S2‐FigureS1‐S51.docx. ^1^H‐NMR spectrum of compound **3b‐I**.
**Figure S2:** ^13^C‐NMR spectrum of compound **3b‐I**.
**Figure S3:** ^1^H‐NMR spectrum of compound **3d‐I**.
**Figure S4:** ^13^C‐NMR spectrum of compound **3d‐I**.
**Figure S5:** ^1^H‐NMR spectrum of compound **3b‐II**.
**Figure S6:** ^13^C‐NMR spectrum of compound **3b‐II**.
**Figure S7:** ^1^H‐NMR spectrum of compound **3c‐II**.
**Figure S8:** ^13^C‐NMR spectrum of compound **3c‐II**.
**Figure S9:** ^1^H‐NMR spectrum of compound **3d‐II**.
**Figure S10:** ^13^C‐NMR spectrum of compound **3d‐II**.
**Figure S11:** ^1^H‐NMR spectrum of compound **3g‐II**.
**Figure S12:** ^13^C‐NMR spectrum of compound **3g‐II**.
**Figure S13:** ^1^H‐NMR spectrum of compound **5c‐II**.
**Figure S14:** ^13^C‐NMR spectrum of compound **5c‐II**.
**Figure S15:** ^1^H‐NMR spectrum of compound **5f‐II**.
**Figure S16:** ^13^C‐NMR spectrum of compound **5f‐II**.
**Figure S17:** cbdd70353‐sup‐0001‐TableS1‐S2‐FigureS1‐S51.docx. ^1^H‐NMR spectrum of compound **6c‐II**.
**Figure S18:** cbdd70353‐sup‐0001‐TableS1‐S2‐FigureS1‐S51.docx. ^13^C‐NMR spectrum of compound **6c‐II**.
**Figure S19:** ^1^H‐NMR spectrum of compound **3c‐I**.
**Figure S20:** ^13^C‐NMR spectrum of compound **3c‐I**.
**Figure S21:** ^1^H‐NMR spectrum of compound **3f‐II**.
**Figure S22:** ^13^C‐NMR spectrum of compound **3f‐II**.
**Figure S23:** ^1^H‐NMR spectrum of compound **3h‐II**.
**Figure S24:** ^13^C‐NMR spectrum of compound **3h‐II**.
**Figure S25:** HRMS (ESI+) spectrum of compound **3b‐I**.
**Figure S26:** HRMS (ESI+) spectrum of compound **3c‐I**.
**Figure S27:** HRMS (ESI+) spectrum of compound **3d‐I**.
**Figure S28:** HRMS (ESI+) spectrum of compound **3b‐II**.
**Figure S29:** HRMS (ESI+) spectrum of compound **3c‐II**.
**Figure S30:** HRMS (ESI+) spectrum of compound **3d‐II**.
**Figure S31:** HRMS (ESI+) spectrum of compound **3f‐II**.
**Figure S32:** HRMS (ESI+) spectrum of compound **3g‐II**.
**Figure S33:** HRMS (ESI+) spectrum of compound **3h‐II**.
**Figure S34:** HRMS (ESI+) spectrum of compound **5c‐II**.
**Figure S35:** HRMS (ESI+) spectrum of compound **5f‐II**.
**Figure S36:** HRMS (ESI+) spectrum of compound **6c‐II**.
**Figure S37:** HPLC chromatogram for compound **3b‐I**.
**Figure S38:** HPLC chromatogram for compound **3c‐I**.
**Figure S39:** HPLC chromatogram for compound **3d‐I**.
**Figure S40:** HPLC chromatogram for compound **3b‐II**.
**Figure S41:** HPLC chromatogram for compound **3c‐II**.
**Figure S42:** HPLC chromatogram for compound **3d‐II**.
**Figure S43:** HPLC chromatogram for compound **3f‐II**.
**Figure S44:** HPLC chromatogram for compound **3g‐II**.
**Figure S45:** HPLC chromatogram for compound **3h‐II**.
**Figure S46:** HPLC chromatogram for compound **5c‐II**.
**Figure S47:** HPLC chromatogram for compound **5f‐II**.
**Figure S48:** HPLC chromatogram for compound **6c‐II**.
**Figure S49:** Distribution of the compounds in the BOILED‐Egg plot with some representative compounds indicated. The yellow region indicates compounds predicted to be permeable through both the gastrointestinal tract (GIT) and the blood–brain barrier (BBB), whereas the white region represents compounds predicted to be permeable only through the GIT. Blue dots indicate compounds predicted to be substrates of P‐glycoprotein, while red dots represent non‐substrates. Selected representative compounds are highlighted.
**Figure S50:** PCA analyses of the compounds from set I and set II using (A) RDKit fingerprints and (B) Layered fingerprints.
**Figure S51:** Flowchat representation of the decision trees to discriminate the compounds from (A) set I and (B) set II.
**Table S1:** Physicochemical and drug‐like properties of the compounds.
**Table S2:** Values for the descriptors selected from decision tree method.

## Data Availability

The data that supports the findings of this study are available in the Supporting Information of this article.
