# Supplementary material for: Probing Cinnamamides and Benzamides as Anthelmintics: Discovery of Potent Drug‐Like Agents Against Angiostrongylus cantonensis
Source: Chem Biol Drug Des. 2026 Jul 4;108(1):e70353. doi: 10.1111/cbdd.70353 (PMC13332563; doi:10.1111/cbdd.70353)
Supplement: Supplementary file 1 — Figure S1: cbdd70353‐sup‐0001‐TableS1‐S2‐FigureS1‐S51.docx. 1H‐NMR spectrum of compound 3b‐I. Figure S2: 13C‐NMR spectrum of compound 3b‐I. Figure S3: 1H‐NMR spectrum of compound 3d‐I. Figure S4: 13C‐NMR spectrum of compound 3d‐I. Figure S5: 1H‐NMR spectrum of compound 3b‐II. Figure S6: 13C‐NMR spectrum of compound 3b‐II. Figure S7: 1H‐NMR spectrum of compound 3c‐II. Figure S8: 13C‐NMR spectrum of compound 3c‐II. Figure S9: 1H‐NMR spectrum of compound 3d‐II. Figure S10: 13C‐NMR spectrum of compound 3d‐II. Figure S11: 1H‐NMR spectrum of compound 3g‐II. Figure S12: 13C‐NMR spectrum of compound 3g‐II. Figure S13: 1H‐NMR spectrum of compound 5c‐II. Figure S14: 13C‐NMR spectrum of compound 5c‐II. Figure S15: 1H‐NMR spectrum of compound 5f‐II. Figure S16: 13C‐NMR spectrum of compound 5f‐II. Figure S17: cbdd70353‐sup‐0001‐TableS1‐S2‐FigureS1‐S51.docx. 1H‐NMR spectrum of compound 6c‐II. Figure S18: cbdd70353‐sup‐0001‐TableS1‐S2‐FigureS1‐S51.docx. 13C‐NMR spectrum of compound 6c‐II. Figure S19: 1H‐NMR spectrum of compound 3c‐I. Figure S20: 13C‐NMR spectrum of compound 3c‐I. Figure S21: 1H‐NMR spectrum of compound 3f‐II. Figure S22: 13C‐NMR spectrum of compound 3f‐II. Figure S23: 1H‐NMR spectrum of compound 3h‐II. Figure S24: 13C‐NMR spectrum of compound 3h‐II. Figure S25: HRMS (ESI+) spectrum of compound 3b‐I. Figure S26: HRMS (ESI+) spectrum of compound 3c‐I. Figure S27: HRMS (ESI+) spectrum of compound 3d‐I. Figure S28: HRMS (ESI+) spectrum of compound 3b‐II. Figure S29: HRMS (ESI+) spectrum of compound 3c‐II. Figure S30: HRMS (ESI+) spectrum of compound 3d‐II. Figure S31: HRMS (ESI+) spectrum of compound 3f‐II. Figure S32: HRMS (ESI+) spectrum of compound 3g‐II. Figure S33: HRMS (ESI+) spectrum of compound 3h‐II. Figure S34: HRMS (ESI+) spectrum of compound 5c‐II. Figure S35: HRMS (ESI+) spectrum of compound 5f‐II. Figure S36: HRMS (ESI+) spectrum of compound 6c‐II. Figure S37: HPLC chromatogram for compound 3b‐I. Figure S38: HPLC chromatogram for compound 3c‐I. Figure S3 [file CBDD-108-e70353-s001.docx]

**PROBING CINNAMAMIDES AND BENZAMIDES AS ANTHELMINTICS: DISCOVERY OF POTENT DRUG-LIKE AGENTS AGAINST *Angiostrongylus cantonensis***

Running title: Cinnamamides and benzamides as anthelmintics

**Bruna L. Lemes^1‡^, Mariana A. Siegl-Breno^2‡^, Marina T. Varela^2,3^, Flavia B. Lopes^2,3^, Mikaelly K. Silva-Nunes^4^, Thaiane D. Santos^2^, Lucas Fukui-Silva^1^, Daniel B. Roquini^1^, Vinicius G. Maltarollo^5^, Josué de Moraes^1,4^* and João Paulo S. Fernandes^2^***

^1^Research Center on Neglected Diseases, Guarulhos University, Guarulhos, Brazil

^2^Department of Pharmaceutical Sciences, Federal University of São Paulo, Diadema, Brazil

^3^Department of Medicine, Federal University of São Paulo, São Paulo, Brazil

^4^Research Center on Neglected Diseases, Scientific and Technological Institute, Brasil University, São Paulo, Brazil

^5^Department of Pharmaceutical Products, Faculty of Pharmacy, Federal University of Minas Gerais, Belo Horizonte, Brazil

^‡^These authors contributed equally

*Corresponding authors: moraesnpdn@gmail.com (JdM); joao.fernandes@unifesp.br (JPSF)

**Supporting Information**

NMR spectra of the synthesized compounds S2-S13

HRMS spectra of the synthesized compounds S14-S17

HPLC chromatograms of the synthesized compounds ………………………...S18-S21

Supplementary Tables S1 and S2 S22-S23

Supplementary Figures S49-S51 S24-S25

**Figure S1.** ^1^H-NMR spectrum of compound **3b-I**

**Figure S2.** ^13^C-NMR spectrum of compound **3b-I**

**Figure S3.** ^1^H-NMR spectrum of compound **3d-I**

**Figure S4.** ^13^C-NMR spectrum of compound **3d-I**

**Figure S5.** ^1^H-NMR spectrum of compound **3b-II**

**Figure S6.** ^13^C-NMR spectrum of compound **3b-II**

**Figure S7.** ^1^H-NMR spectrum of compound **3c-II**

**Figure S8.** ^13^C-NMR spectrum of compound **3c-II**

**Figure S9.** ^1^H-NMR spectrum of compound **3d-II**

**Figure S10.** ^13^C-NMR spectrum of compound **3d-II**

**Figure S11.** ^1^H-NMR spectrum of compound **3g-II**

**Figure S12.** ^13^C-NMR spectrum of compound **3g-II**

**Figure S13.** ^1^H-NMR spectrum of compound **5c-II**

**Figure S14.** ^13^C-NMR spectrum of compound **5c-II**

**Figure S15.** ^1^H-NMR spectrum of compound **5f-II**

**Figure S16.** ^13^C-NMR spectrum of compound **5f-II**

**Figure S17.** ^1^H-NMR spectrum of compound **6c-II**

**Figure S18.** ^13^C-NMR spectrum of compound **6c-II**

**Figure S19.** ^1^H-NMR spectrum of compound **3c-I**

**Figure S20.** ^13^C-NMR spectrum of compound **3c-I**

**Figure S21.** ^1^H-NMR spectrum of compound **3f-II**

**Figure S22.** ^13^C-NMR spectrum of compound **3f-II**

**Figure S23.** ^1^H-NMR spectrum of compound **3h-II**

**Figure S24.** ^13^C-NMR spectrum of compound **3h-II**

**Figure S25.** HRMS (ESI+) spectrum of compound **3b-I**

**Figure S26.** HRMS (ESI+) spectrum of compound **3c-I**

**Figure S27.** HRMS (ESI+) spectrum of compound **3d-I**

**Figure S28.** HRMS (ESI+) spectrum of compound **3b-II**

**Figure S29.** HRMS (ESI+) spectrum of compound **3c-II**

**Figure S30.** HRMS (ESI+) spectrum of compound **3d-II**

**Figure S31.** HRMS (ESI+) spectrum of compound **3f-II**

**Figure S32.** HRMS (ESI+) spectrum of compound **3g-II**

**Figure S33.** HRMS (ESI+) spectrum of compound **3h-II**

**Figure S34.** HRMS (ESI+) spectrum of compound **5c-II**

**Figure S35.** HRMS (ESI+) spectrum of compound **5f-II**

**Figure S36.** HRMS (ESI+) spectrum of compound **6c-II**


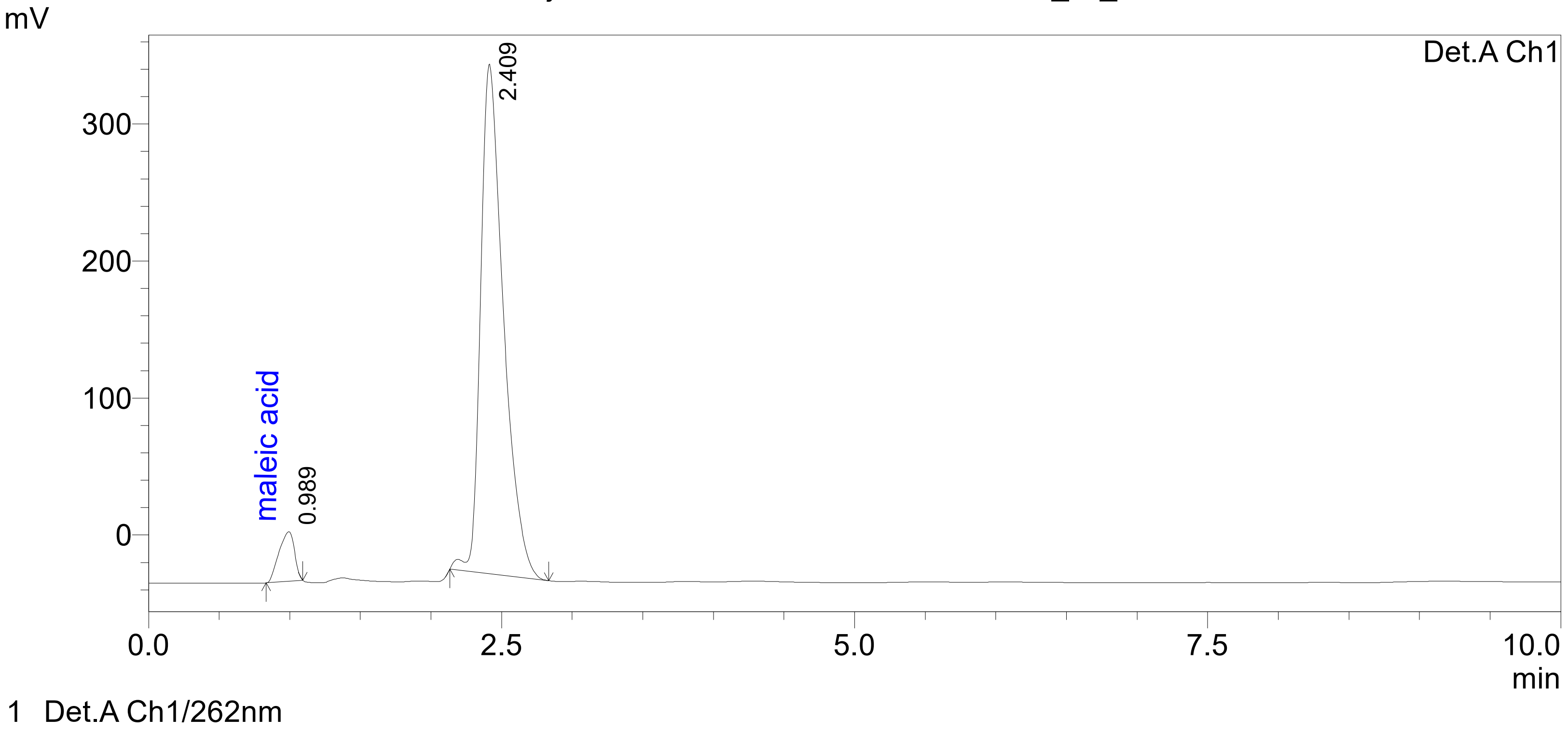


**Figure S37.** HPLC chromatogram for compound **3b-I**


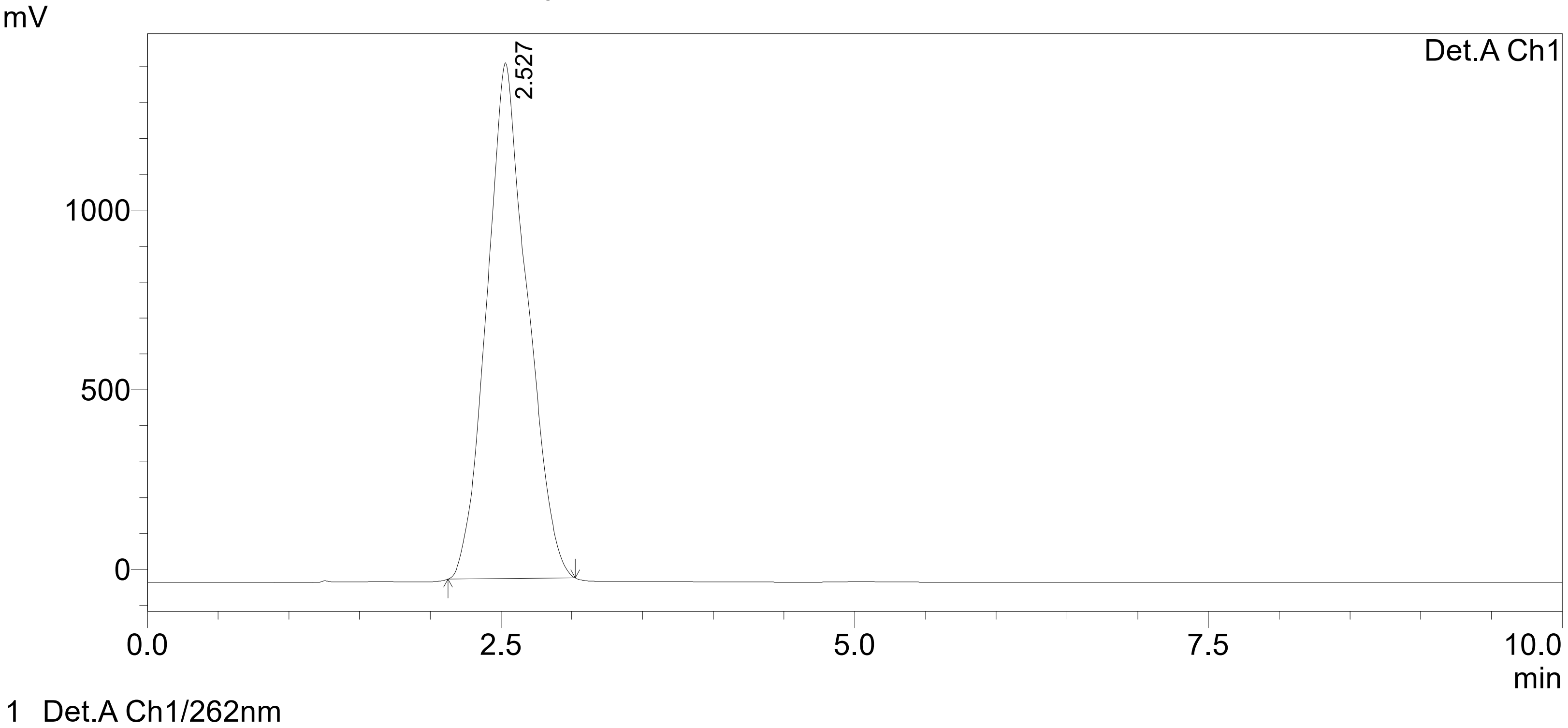


**Figure S38.** HPLC chromatogram for compound **3c-I**


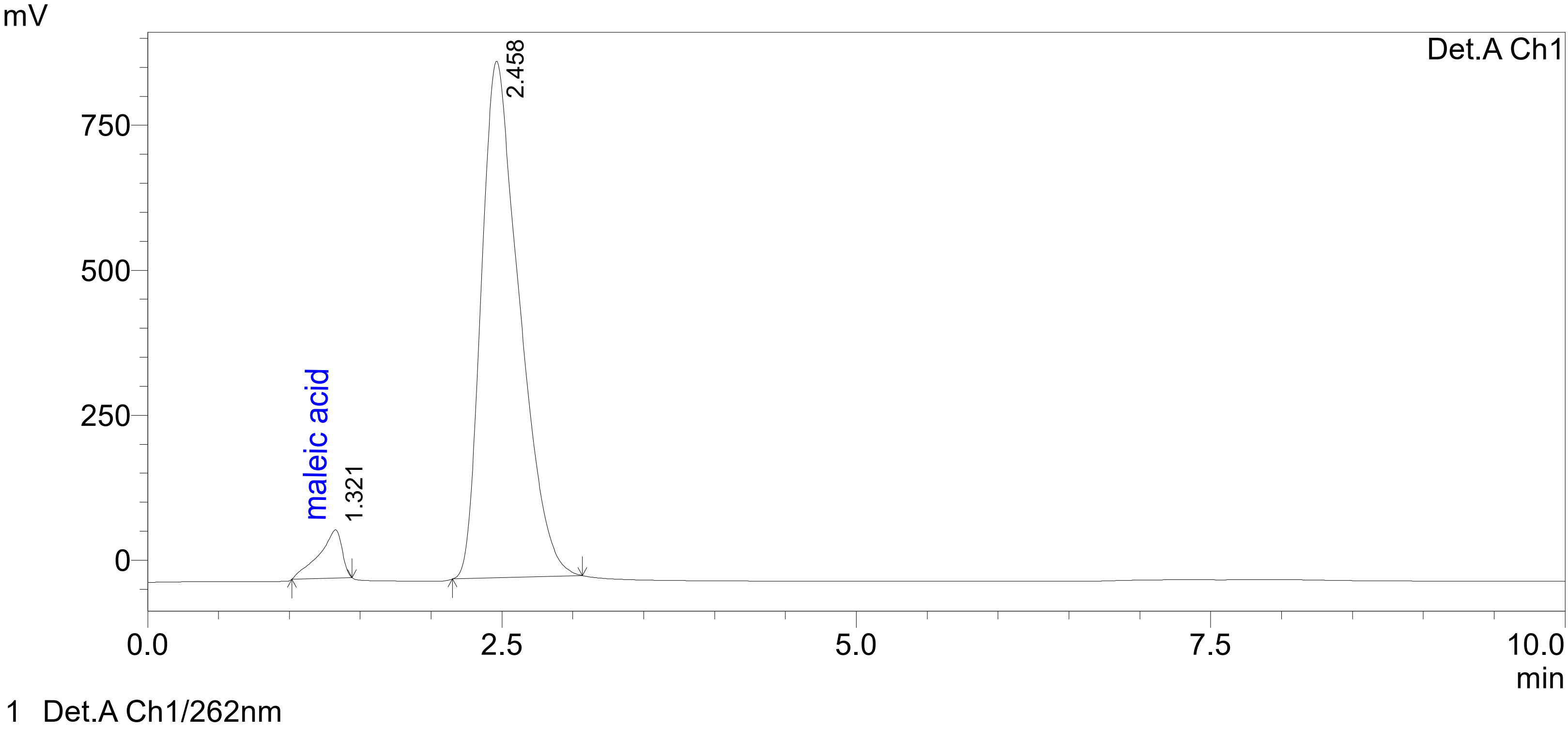


**Figure S39.** HPLC chromatogram for compound **3d-I**


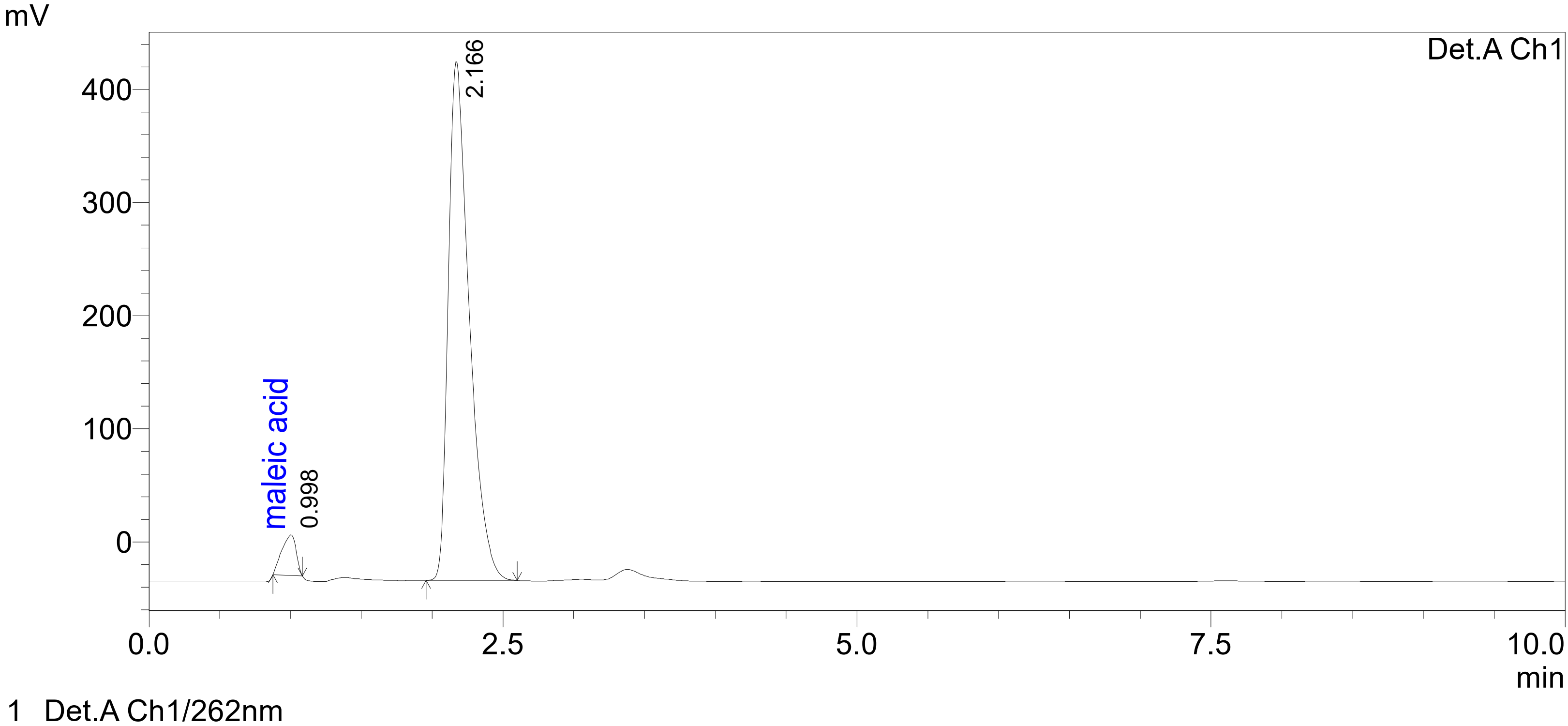


**Figure S40.** HPLC chromatogram for compound **3b-II**


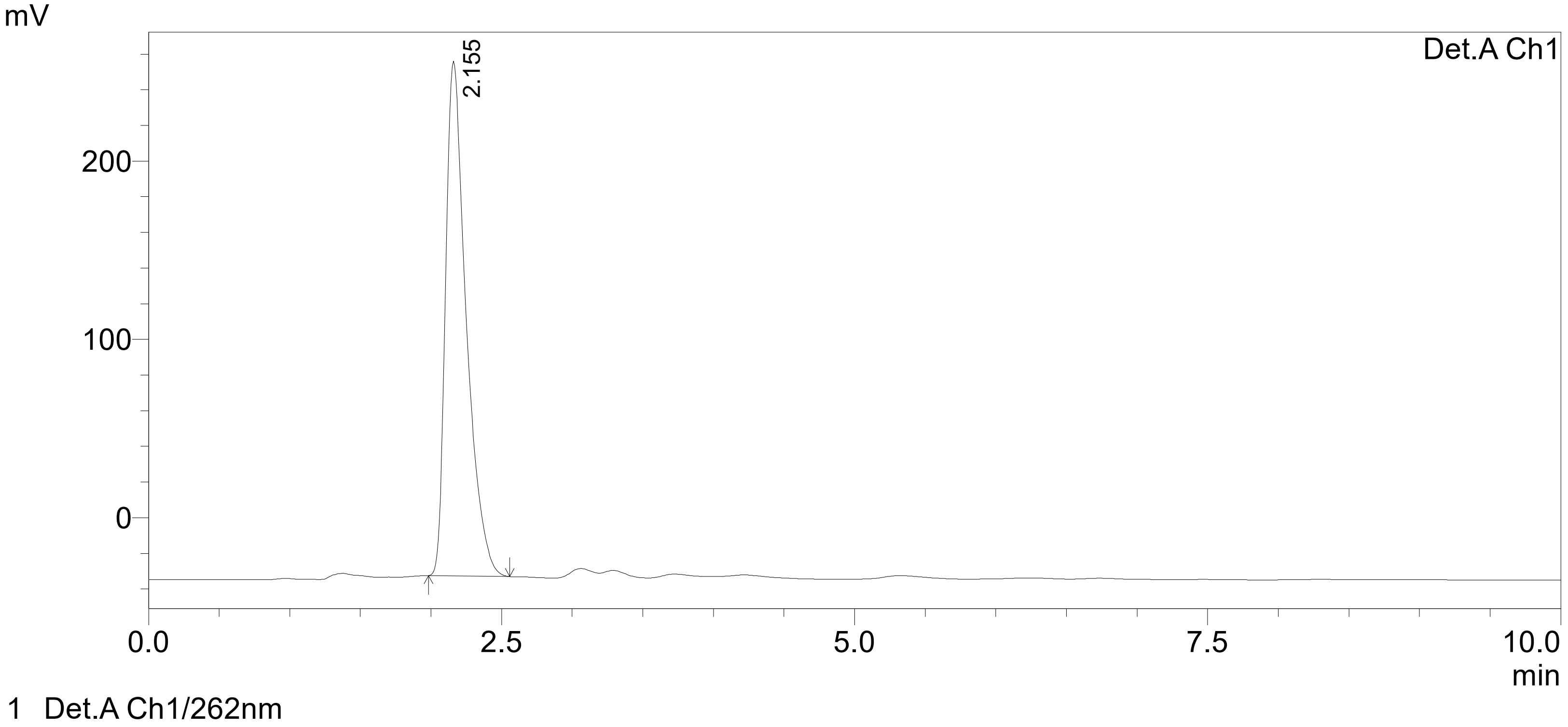


**Figure S41.** HPLC chromatogram for compound **3c-II**


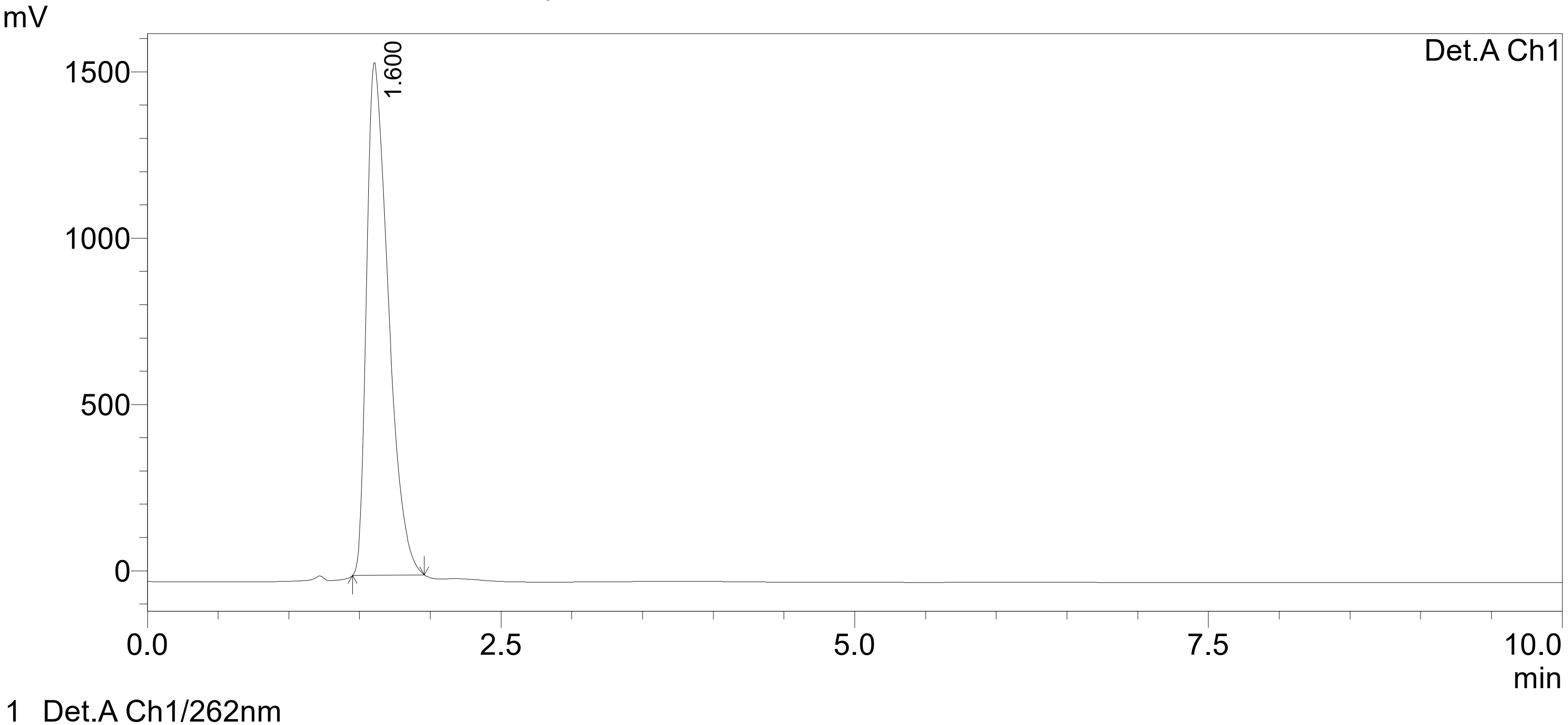


**Figure S42.** HPLC chromatogram for compound **3d-II**


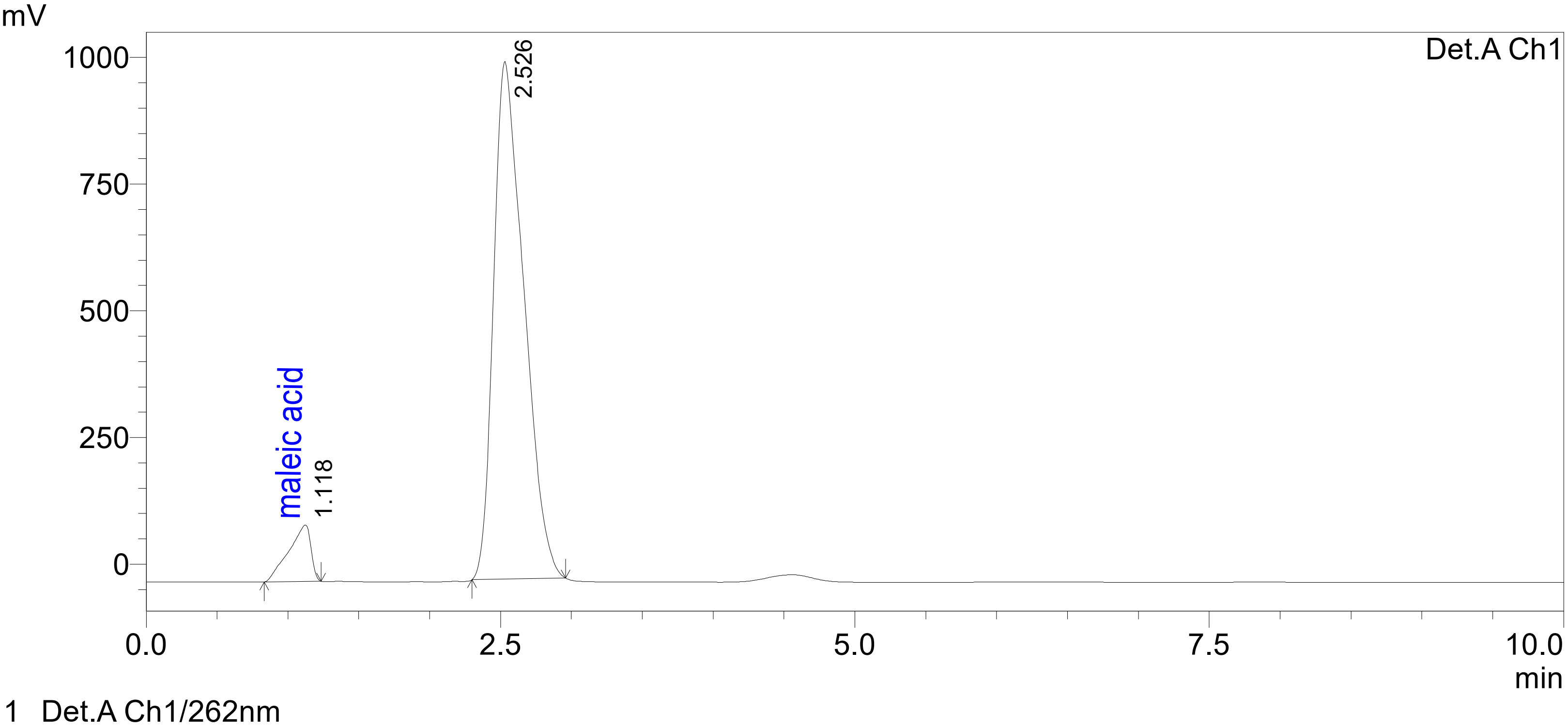


**Figure S43.** HPLC chromatogram for compound **3f-II**


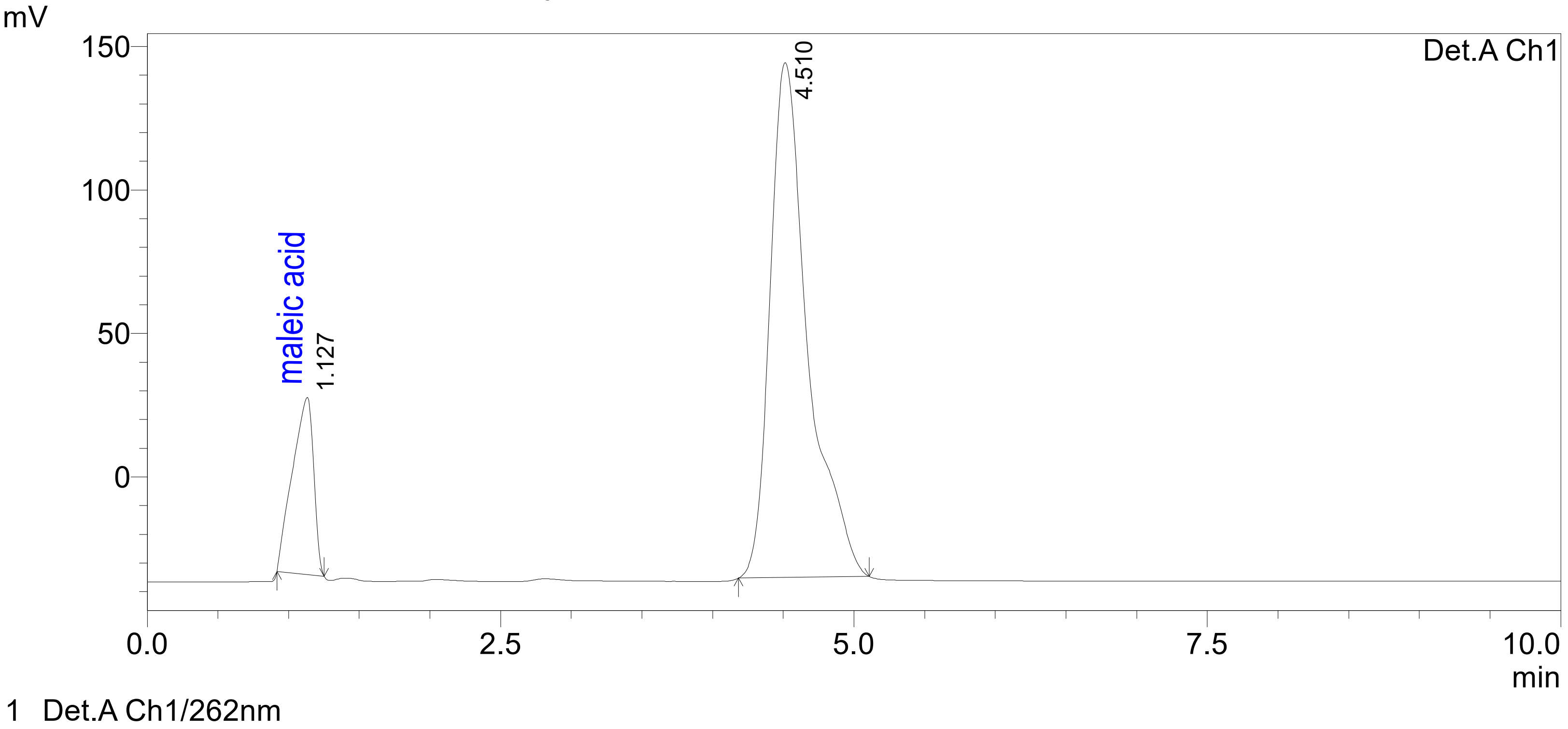


**Figure S44.** HPLC chromatogram for compound **3g-II**


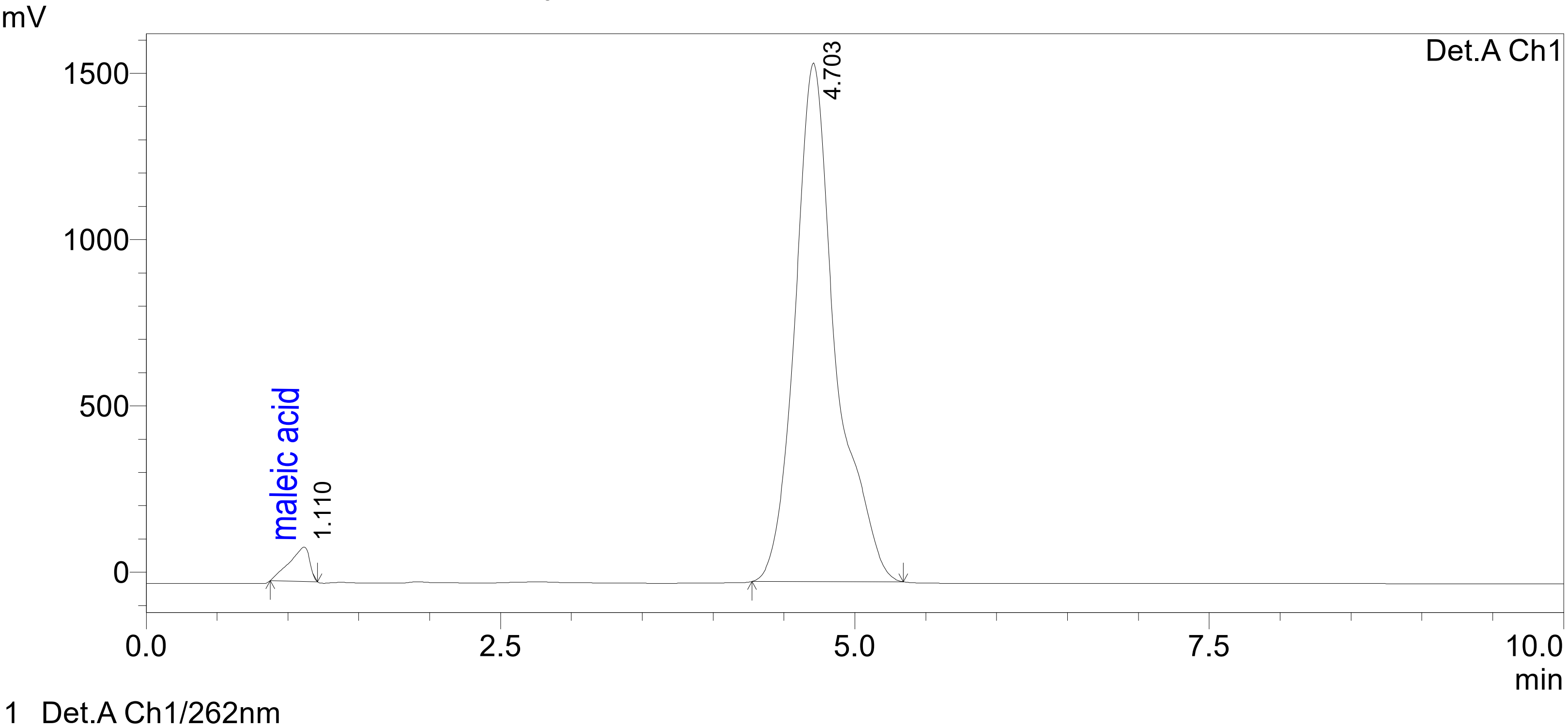


**Figure S45.** HPLC chromatogram for compound **3h-II**


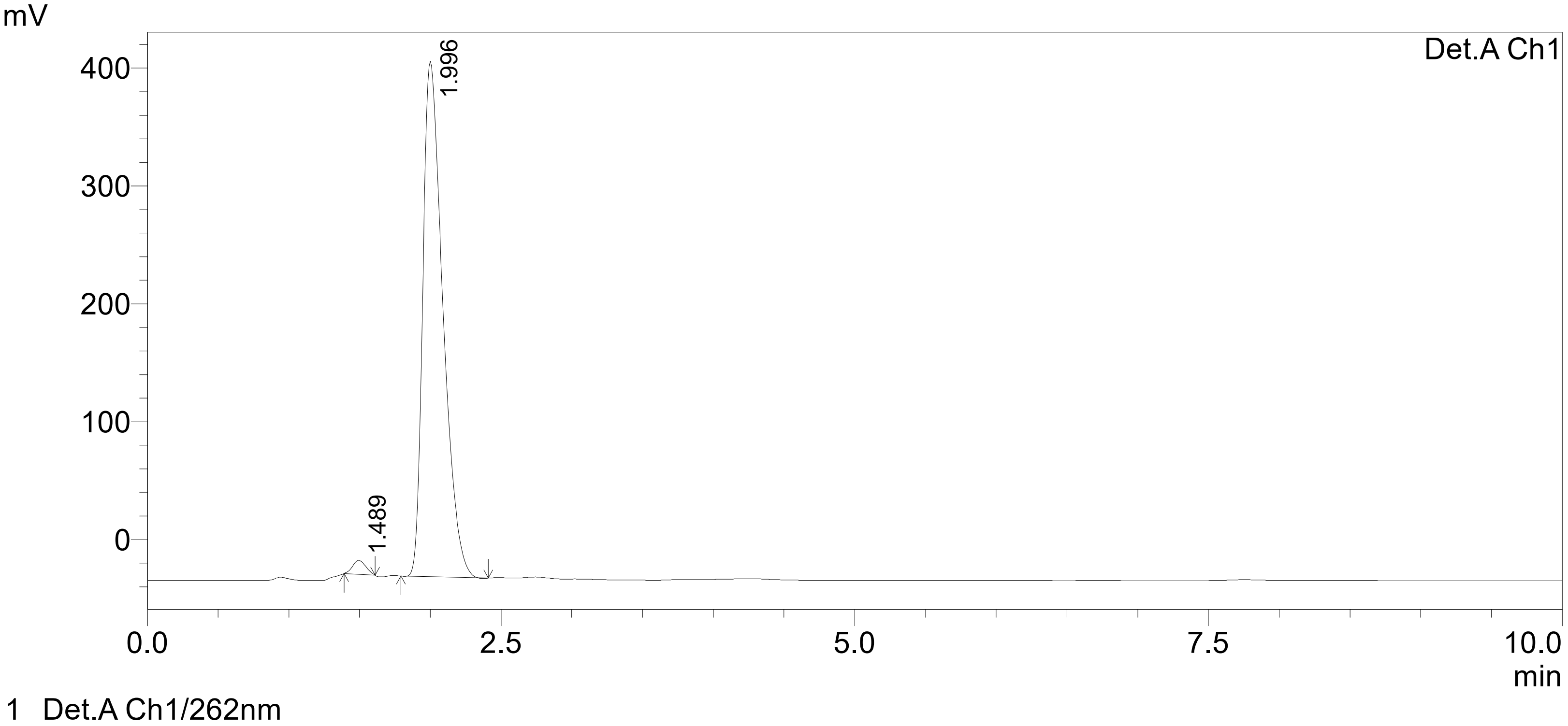


**Figure S46.** HPLC chromatogram for compound **5c-II**


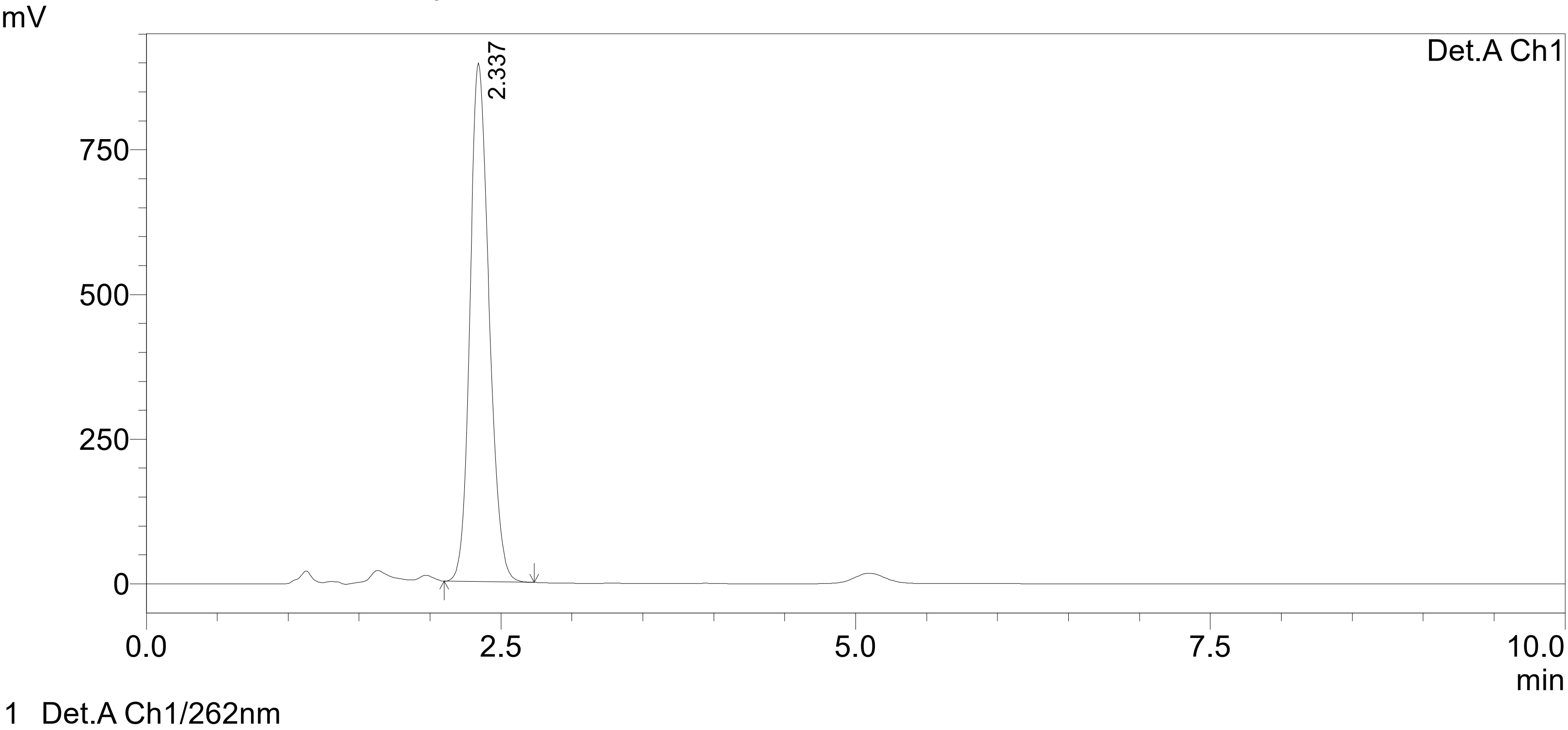


**Figure S47.** HPLC chromatogram for compound **5f-II**


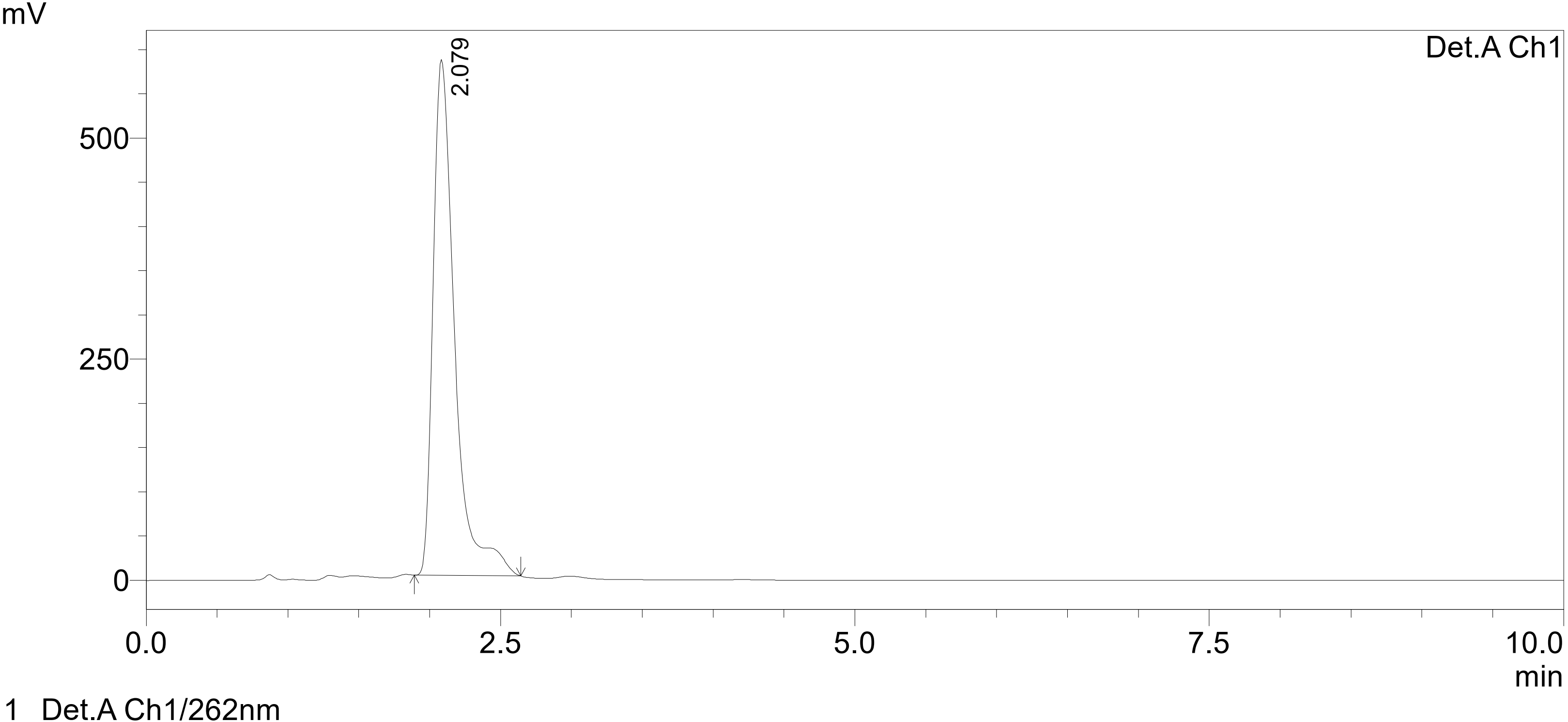


**Figure S48.** HPLC chromatogram for compound **6c-II**

**Table S1.** Physicochemical and drug-like properties of the compounds.

| **ID** | **MW** | **Fsp3** | **RB** | **HBA** | **HBD** | **MR** | **TPSA** | **WLOGP** | **Log P** | **LogS** | **LogKp (cm/s)** | **GI absorption** | **BBB permeant** | **Pgp substrate** | **QED** |
| --- | --- | --- | --- | --- | --- | --- | --- | --- | --- | --- | --- | --- | --- | --- | --- |
| **1a-I** | 336.43 | 0.29 | 6 | 3 | 0 | 108.06 | 32.78 | 2.03 | 2.95 | -3.83 | -6.17 | High | Yes | No | 0.787 |
| **1b-I** | 322.40 | 0.25 | 5 | 3 | 1 | 103.59 | 43.78 | 1.73 | 2.54 | -3.62 | -6.31 | High | Yes | No | 0.880 |
| **1c-I** | 352.43 | 0.29 | 6 | 4 | 1 | 110.08 | 53.01 | 1.74 | 2.59 | -3.7 | -6.5 | High | Yes | No | 0.841 |
| **1d-I** | 338.40 | 0.25 | 5 | 4 | 2 | 105.62 | 64.01 | 1.43 | 2.16 | -3.48 | -6.66 | High | Yes | Yes | 0.664 |
| **1e-I** | 350.41 | 0.29 | 5 | 4 | 0 | 107.63 | 42.01 | 1.75 | 2.86 | -4.09 | -6.11 | High | Yes | No | 0.795 |
| **2a-I** | 335.44 | 0.32 | 6 | 2 | 0 | 106.06 | 29.54 | 3.7 | 4.02 | -4.7 | -5.17 | High | Yes | No | 0.767 |
| **2b-I** | 321.41 | 0.29 | 5 | 2 | 1 | 101.59 | 40.54 | 3.4 | 3.63 | -4.54 | -5.25 | High | Yes | No | 0.868 |
| **3b-I** | 366.41 | 0.29 | 5 | 5 | 1 | 109.66 | 62.24 | 1.46 | 2.45 | -3.96 | -6.45 | High | Yes | No | 0.843 |
| **3c-I** | 396.44 | 0.32 | 6 | 6 | 1 | 116.15 | 71.47 | 1.46 | 2.37 | -3.81 | -6.91 | High | Yes | Yes | 0.783 |
| **3d-I** | 382.41 | 0.29 | 5 | 6 | 2 | 111.68 | 82.47 | 1.16 | 2.03 | -3.6 | -7.06 | High | No | Yes | 0.623 |
| **4a-I** | 322.40 | 0.25 | 5 | 2 | 0 | 103.81 | 32.78 | 2.19 | 2.88 | -4.00 | -5.87 | High | Yes | No | 0.811 |
| **4c-I** | 338.40 | 0.25 | 5 | 3 | 1 | 105.84 | 53.01 | 1.89 | 2.44 | -3.88 | -6.21 | High | Yes | No | 0.871 |
| **4d-I** | 324.37 | 0.21 | 4 | 3 | 2 | 101.37 | 64.01 | 1.59 | 2.09 | -3.65 | -6.37 | High | Yes | No | 0.672 |
| **1a-II** | 310.39 | 0.32 | 5 | 3 | 0 | 98.35 | 32.78 | 1.74 | 2.40 | -2.97 | -6.9 | High | Yes | Yes | 0.870 |
| **1b-II** | 296.36 | 0.28 | 4 | 3 | 1 | 93.88 | 43.78 | 1.44 | 2.00 | -2.76 | -7.05 | High | Yes | Yes | 0.945 |
| **1c-II** | 326.39 | 0.32 | 5 | 4 | 1 | 100.37 | 53.01 | 1.45 | 2.22 | -3.35 | -6.66 | High | Yes | No | 0.937 |
| **1d-II** | 312.36 | 0.28 | 4 | 4 | 2 | 95.91 | 64.01 | 1.14 | 1.79 | -3.15 | -6.8 | High | Yes | Yes | 0.852 |
| **1g-II** | 314.81 | 0.28 | 4 | 2 | 0 | 96.87 | 23.55 | 2.38 | 2.96 | -3.49 | -6.47 | High | Yes | No | 0.868 |
| **2a-II** | 309.40 | 0.35 | 5 | 2 | 0 | 96.35 | 29.54 | 3.41 | 3.70 | -4.50 | -5.16 | High | Yes | No | 0.860 |
| **3b-II** | 340.37 | 0.32 | 4 | 5 | 1 | 99.95 | 62.24 | 1.17 | 2.03 | -3.40 | -6.85 | High | Yes | Yes | 0.927 |
| **3c-II** | 370.40 | 0.35 | 5 | 6 | 1 | 106.44 | 71.47 | 1.17 | 2.08 | -3.47 | -7.05 | High | No | Yes | 0.889 |
| **3d-II** | 356.37 | 0.32 | 4 | 6 | 2 | 101.97 | 82.47 | 0.87 | 1.66 | -3.25 | -7.2 | High | No | Yes | 0.816 |
| **3f-II** | 374.82 | 0.32 | 4 | 5 | 1 | 104.96 | 62.24 | 1.82 | 2.58 | -3.99 | -6.62 | High | Yes | Yes | 0.895 |
| **3g-II** | 358.82 | 0.32 | 4 | 4 | 0 | 102.93 | 42.01 | 2.11 | 2.95 | -4.13 | -6.27 | High | Yes | No | 0.845 |
| **3h-II** | 364.39 | 0.29 | 4 | 5 | 0 | 107.69 | 55.15 | 2.21 | 2.82 | -4.29 | -6.22 | High | Yes | Yes | 0.715 |
| **4a-II** | 296.36 | 0.28 | 4 | 2 | 0 | 94.10 | 32.78 | 1.90 | 2.54 | -3.68 | -6.01 | High | Yes | No | 0.873 |
| **4c-II** | 312.36 | 0.28 | 4 | 3 | 1 | 96.13 | 53.01 | 1.60 | 2.24 | -3.76 | -6.1 | High | Yes | No | 0.945 |
| **4d-II** | 298.34 | 0.24 | 3 | 3 | 2 | 91.66 | 64.01 | 1.300 | 1.73 | -3.32 | -6.52 | High | Yes | Yes | 0.833 |
| **5c-II** | 342.39 | 0.32 | 5 | 4 | 1 | 102.62 | 62.24 | 1.61 | 2.15 | -3.60 | -6.57 | High | Yes | No | 0.924 |
| **5f-II** | 346.81 | 0.28 | 4 | 3 | 1 | 101.14 | 53.01 | 2.25 | 2.68 | -4.12 | -6.13 | High | Yes | No | 0.928 |
| **6c-II** | 342.39 | 0.32 | 5 | 4 | 1 | 102.62 | 62.24 | 1.61 | 2.16 | -3.60 | -6.57 | High | Yes | No | 0.924 |
| **ALB** | 265.33 | 0.33 | 6 | 3 | 2 | 73.22 | 92.31 | 3.05 | 2.48 | -3.23 | -5.92 | High | No | No | 0.833 |

**Table S2.** Values for the descriptors selected from decision tree method.

| **ID** | **VE1_Dzi** | **GATS8i** | **AATSC2c** |
| --- | --- | --- | --- |
| **1a-I** | 0.01473 | 1.02625 | -0.00097 |
| **1b-I** | 0.01956 | 1.02331 | -0.00030 |
| **1c-I** | 0.08435 | 1.07353 | -0.00155 |
| **1d-I** | 0.06133 | 1.01068 | -0.00089 |
| **1e-I** | 0.09011 | 1.06359 | -0.00096 |
| **2a-I** | 0.07908 | 0.97452 | 0.00002 |
| **2b-I** | 0.08207 | 0.96888 | 0.00073 |
| **3b-I** | 0.06028 | 1.08192 | -0.00042 |
| **3c-I** | 0.00441 | 1.12489 | -0.00156 |
| **3d-I** | 0.01987 | 1.07043 | -0.00096 |
| **4a-I** | 0.05719 | 1.04672 | -0.00045 |
| **4c-I** | 0.01697 | 1.10119 | -0.00108 |
| **4d-I** | 0.00736 | 1.02942 | -0.00033 |
| **1a-II** | 0.06996 | 0.73253 | -0.00153 |
| **1b-II** | 0.08174 | 0.83840 | -0.00085 |
| **1c-II** | 0.14261 | 0.96735 | -0.00198 |
| **1d-II** | 0.12351 | 0.82516 | -0.00130 |
| **1g-II** | 0.07977 | 0.78955 | -0.00126 |
| **2a-II** | 0.13010 | 0.76197 | -0.00046 |
| **3b-II** | 0.00879 | 0.93440 | -0.00093 |
| **3c-II** | 0.05528 | 1.03883 | -0.00195 |
| **3d-II** | 0.03293 | 0.91739 | -0.00133 |
| **3f-II** | 0.03153 | 0.96094 | -0.00140 |
| **3g-II** | 0.01047 | 0.89497 | -0.00130 |
| **3h-II** | 0.10037 | 1.10372 | -0.00238 |
| **4a-II** | 0.00999 | 0.58202 | -0.00101 |
| **4c-II** | 0.06726 | 0.88518 | -0.00151 |
| **4d-II** | 0.04953 | 0.69974 | -0.00073 |
| **5c-II** | 0.07486 | 1.01394 | -0.00149 |
| **5f-II** | 0.05525 | 0.91471 | -0.00084 |
| **6c-II** | 0.00503 | 0.72307 | -0.00243 |


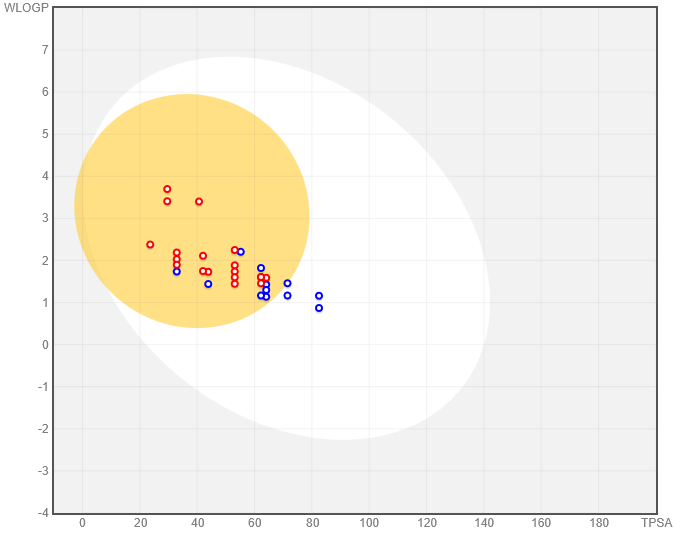


**3c-II**

**3d-II**

**3d-I**

**4a-I**

**1c-II**

**5c-II**

**Figure S49.** Distribution of the compounds in the BOILED-Egg plot with some representative compounds indicated. The yellow region indicates compounds predicted to be permeable through both the gastrointestinal tract (GIT) and the blood–brain barrier (BBB), whereas the white region represents compounds predicted to be permeable only through the GIT. Blue dots indicate compounds predicted to be substrates of P-glycoprotein, while red dots represent non-substrates. Selected representative compounds are highlighted.


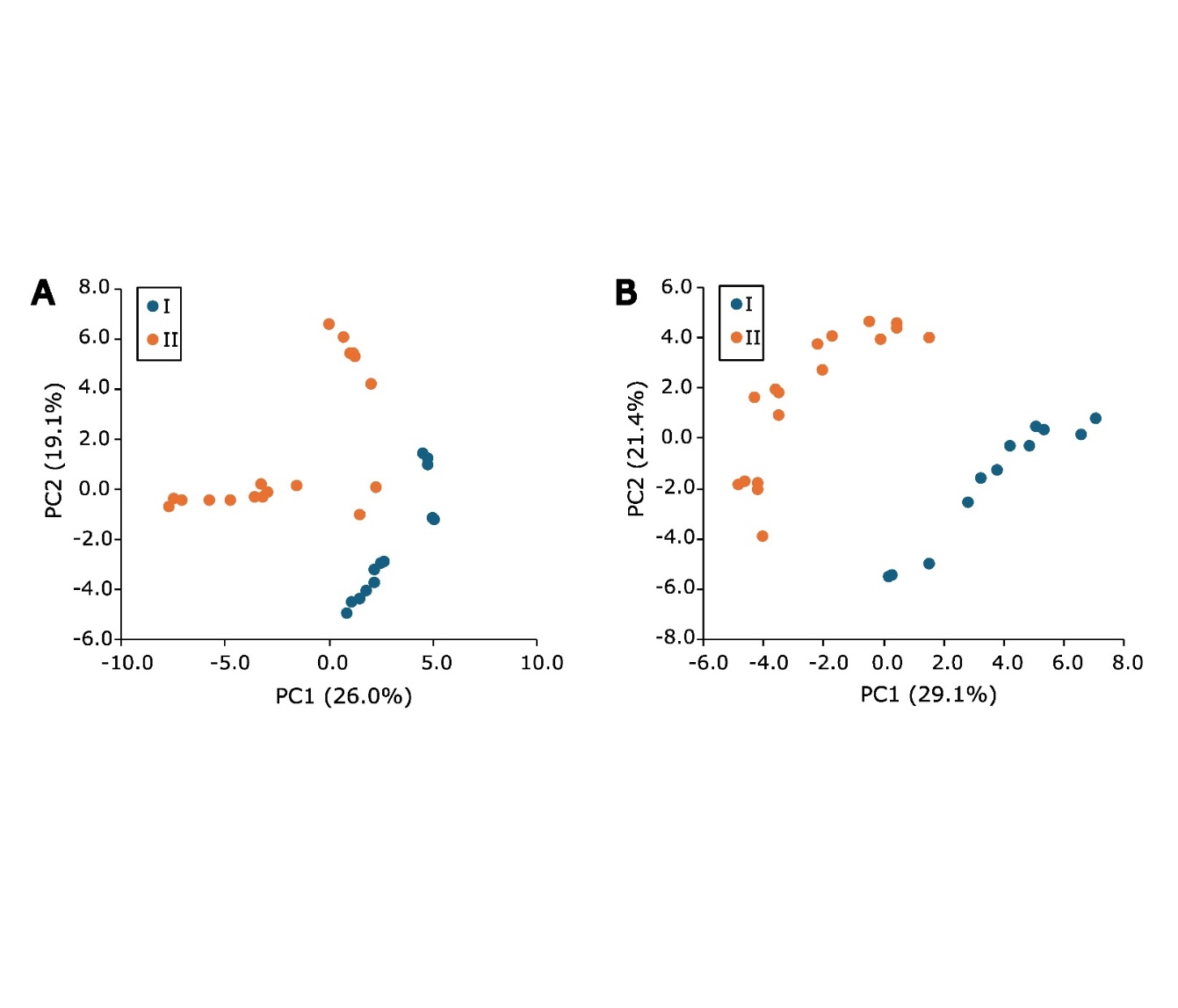


**Figure S50.** PCA analyses of the compounds from set I and set II using (A) RDKit fingerprints and (B) Layered fingerprints.


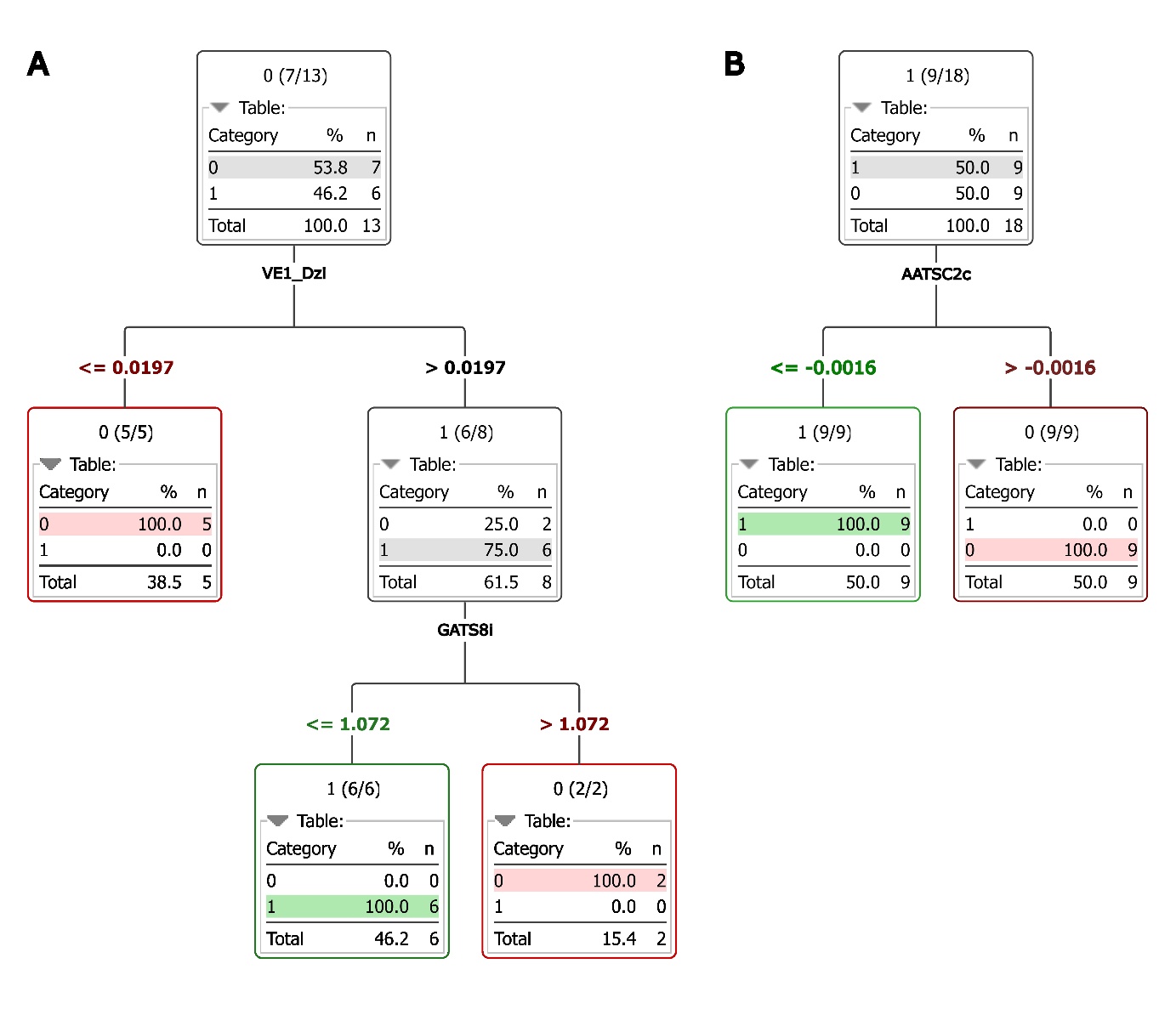


**Figure S51.** Flowchat representation of the decision trees to discriminate the compounds from (A) set I and (B) set II.
